# Orally Administered Antibiotics Vancomycin and Ampicillin Cause Cognitive Impairment With Gut Dysbiosis in Mice With Transient Global Forebrain Ischemia

**DOI:** 10.3389/fmicb.2020.564271

**Published:** 2020-11-26

**Authors:** Kyung-Eon Lee, Jeon-Kyung Kim, Dong-Hyun Kim

**Affiliations:** Department of Life and Nanopharmaceutical Sciences, Neurobiota Research Center, College of Pharmacy, Kyung Hee University, Seoul, South Korea

**Keywords:** antibacterials, brain ischemia, memory, gut dysbiosis, fecal transplantation, *Enterobacter xiangfangenesis*

## Abstract

Gut microbiota is closely associated with the occurrence of neuropsychiatric disorders. Antibiotics are frequently used to prevent pathogen infection in patients with brain ischemia. To understand the impact of prophylactic antibiotic treatment for patients with brain ischemia, we examined the effects of orally administered vancomycin and ampicillin on cognitive function and gut microbiota composition in mice with transient global forebrain ischemia (tIsc). tIsc operation and orally gavaged vancomycin mildly and moderately caused cognitive impairment, respectively. However, the exposure of mice with tIsc to vancomycin or ampicillin severely impaired cognitive function in the Y-maze, novel object recognition, and Banes maze tasks. Furthermore, their treatments induced NF-κB activation as well as active microglia (NF-κB^+^/Iba1^+^ and LPS^+^/Iba1^+^ cells) and apoptotic (caspase 3^+^/NeuN^+^) cell population in the hippocampus, whereas the brain-derived neurotrophic factor (BDNF)^+^/NeuN^+^ cell populations decreased. These treatments also caused colitis and gut dysbiosis. They increased the population of Proteobacteria including *Enterobacter xiangfangenesis.* Orally delivered fecal transplantation of vancomycin-treated mice with or without tIsc and oral gavage of *Enterobacter xiangfangenesis* also significantly deteriorated the cognitive impairment and colitis in transplanted mice with tIsc. These findings suggest that oral administration of antibiotics can deteriorate cognitive impairment with gut dysbiosis in patients with brain ischemia.

## Introduction

Brain ischemia occurs when there is an insufficient amount of blood flow to the brain (Clemens et al., 1997; Bramlett and Dietrich, 2004). This deprives brain tissue of oxygen and nutrients, resulting in brain cell death, cerebral infarction, or ischemic stroke. Brain inflammation occurs in the damaged brain tissue following the infiltration of immune cells, which secrete proinflammatory cytokines tumor necrosis factor (TNF)-α, interleukin (IL)-1β, and IL-6 in ischemic brain injury, and this leads to the impairment of brain functions such as cognition (Kawabori and Yenari, 2015; Anttila et al., 2017). Microbial infection deteriorates the prognosis of ischemic stroke (Fugate et al., 2014). The prophylactic administration of antibiotics is recommended to reduce the risk of pathogen infections in patients with ischemic brain injury (Enzler et al., 2011; Xi et al., 2017). Therefore, several studies examined the impact of antibiotic treatment on the outcomes in hosts with ischemic brain injury. For example, Benakis et al. (2016) reported that orally administered amoxicillin/clavulanic acid reduces ischemic brain injury in mice by regulating gut microbiota composition, while Dong et al. (2017) reported that orally prophylactic treatment with an antibiotic cocktail consisting of chlortetracycline, penicillin VK, and vancomycin could not reduce microbial infection, and Winek et al. (2016) reported that the depletion of gut microbiota by oral prophylactic treatment with an antibiotic cocktail consisting of ampicillin, vancomycin, ciprofloxacin, imipenem, and metronidazole deteriorates the outcome after murine stroke. Singh et al. (2016) reported that fecal microbiota transplantation of healthy mice into middle cerebral artery (MCA) occlusion-operated mice alleviated the stroke outcome.

Recent studies have indicated that the alteration of gut microbiota by gut environmental factors such as orally administered antibiotics and stressors causes the occurrence of psychiatric disorders with colitis (Lundberg et al., 2016; Ceylani et al., 2018; Cussotto et al., 2018; Jang et al., 2018a). For example, Ceylani et al. (2018) reported that oral administration of ampicillin and cefoperazone caused anxiety and suppressed BDNF expression in mice. Jang et al. (2018a) reported that oral gavage of ampicillin and fecal transplantation of ampicillin-treated mice significantly increased anxiety-like behaviors and caused gut dysbiosis in the transplanted conventional animals. The induction of gut dysbiosis by 2,4,6-trinitrobenzenesulfonic acid (TNBS), a colitis inducer, or immobilization stress increases the lipopolysaccharide (LPS) level in the feces and blood, and inflammation in the colon and hippocampus and suppresses BDNF expression in the hippocampus, resulting in the occurrence of psychiatric disorders (Cani et al., 2008; Deng et al., 2014; Lee et al., 2017; Jang et al., 2018b, c; Rosenzweig et al., 2004). Soto et al. (2018) reported that oral administration of vancomycin reduced brain-derived neurotrophic factor (BDNF) expression and anxiety/depression in mice. These reports suggest that gut dysbiosis is closely associated with the occurrence of psychiatric disorders. Nevertheless, the interplay between antibiotics-induced gut dysbiosis and the occurrence and development of psychiatric disorders in patients with brain ischemia remains unclear.

Therefore, to understand the effects of the antibiotics that are used to prevent pathogen infection on the occurrence of cognitive impairment in patients with brain ischemia, we examined the effects of the orally administered antibiotics vancomycin and ampicillin on cognitive function, gut microbiota composition, and gut and brain inflammation in mice with transient global forebrain ischemia (tIsc).

## Results

### Orally Administered Vancomycin and Ampicillin Caused Cognitive Impairment in Mice With tIsc

**FIGURE 1:**
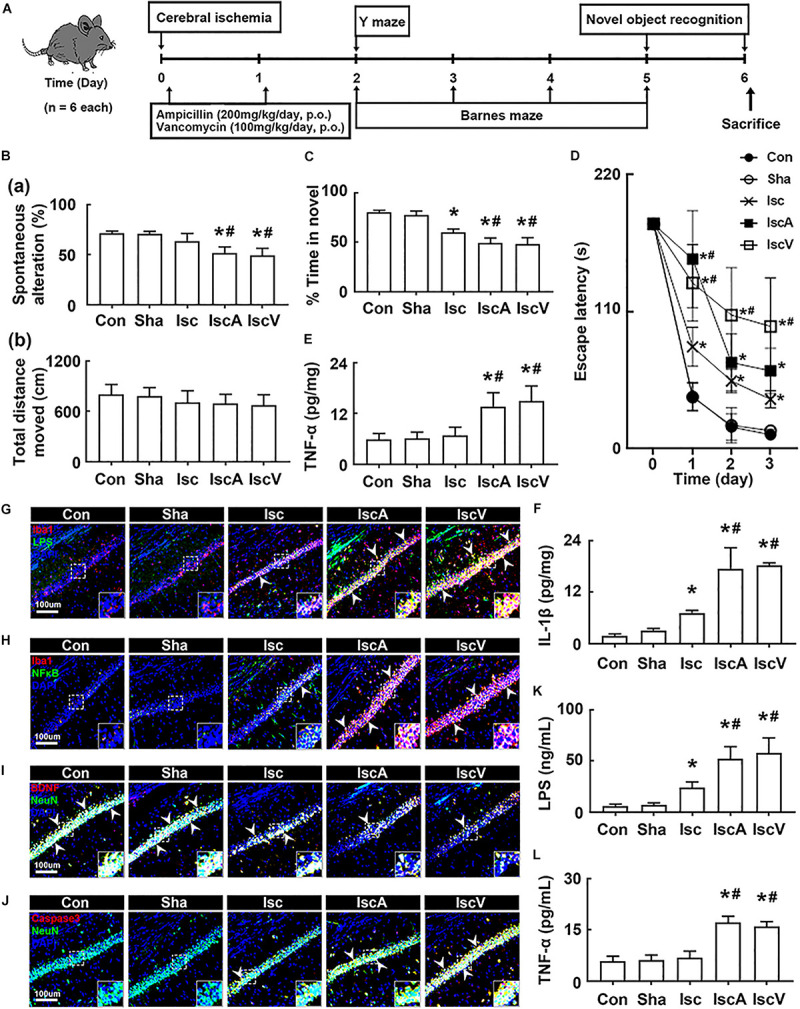
Vancomycin and ampicillin caused the cognitive impairment in mice with and without tIsc. **(A)** Experimental protocol. Effects on the cognitive function, assessed by the Y-maze [**(B)** a, spontaneous alteration; b, moved distance], novel object recognition **(C)**, and Banes maze tasks **(D)**. Effects on TNF-α **(E)** and IL-1β expression **(F)** in the hippocampus, assessed by ELISA. Effects on LPS^+^/Iba1^+^ **(G)**, NF-κB^+^/Iba1^+^ **(H)**, BDNF^+^/NeuN^+^ **(I)**, and caspase 3^+^/NeuN^+^ cell **(J)** populations in the hippocampus, assessed by a confocal microscope. Effects on LPS **(K)** and TNF-α levels **(L)** in the blood. Con, Sha, Isc, IscA, and IscV in the *x*-axis of figures indicate groups treated with vehicle in normal control mice, vehicle in sham mice, vehicle in mice with tIsc, ampicillin in mice with tIsc, and vancomycin in mice with tIsc, respectively. All data were expressed as mean ± SD (*n* = 6). *p < 0.05 vs. Sha group. ^#^*p* < 0.05 vs. Isc group.

First, to understand the effects of the antibiotics used in patients with brain ischemia on the occurrence of cognitive impairment, we induced transient global forebrain ischemia in mice, orally administered ampicillin or vancomycin, and examined cognitive function (Figure 1A). The induction of tIsc itself caused mild cognitive impairment in the Y-maze, novel object recognition maze, and Barnes maze tasks compared to those of control and sham mice. Exposure of mice with tIsc to orally administered vancomycin or ampicillin significantly deteriorated cognitive impairment compared to tIsc mice not treated with antibiotics (Figures 1B–D and Supplementary Figure S1). However, antibiotic treatments did not significantly affect the distance moved in the behavioral tests [the means of arm entries in the Y-maze task and total (target and error) hole visit number in the Barnes maze task] compared to those of the control group treated with the vehicle. Orally administered vancomycin worsened the cognitive function in the Barnes maze task more severely than the orally administered ampicillin. Oral administration of vancomycin or ampicillin increased TNF-α and IL-1β levels (Figures 1E,F). These treatments also increased the Iba1^+^ cell population in the hippocampus of mice with tIsc more strongly than in tIsc mice not treated with antibiotics. In particular, treatment with vancomycin or ampicillin increased the Iba1^+^ cell population in the CA1 region of the hippocampus more than in the CA2 and CA3 regions (Figures 1G,H and Supplementary Figure S2). Furthermore, these antibiotic treatments increased the LPS^+^/Iba1^+^ and NF-κB^+^/Iba1^+^ cell (activated microglia) populations in the CA1 region of the hippocampus of mice with tIsc more strongly than in tIsc mice not treated orally with vancomycin or ampicillin. Furthermore, treatment with antibiotics increased the caspase 3^+^/NeuN^+^ (apoptotic neuron) cell population in the CA1 region of the hippocampus, while the BDNF^+^/NeuN^+^ cell population was suppressed (Figures 1I,J and Supplementary Figure S3). The induction of tIsc increased the blood TNF-α level in mice somewhat, but not significantly, compared to those of control and sham mice. However, orally administered vancomycin or ampicillin markedly increased blood LPS and TNF-α levels in mice with tIsc (Figures 1K,L).

**FIGURE 2:**
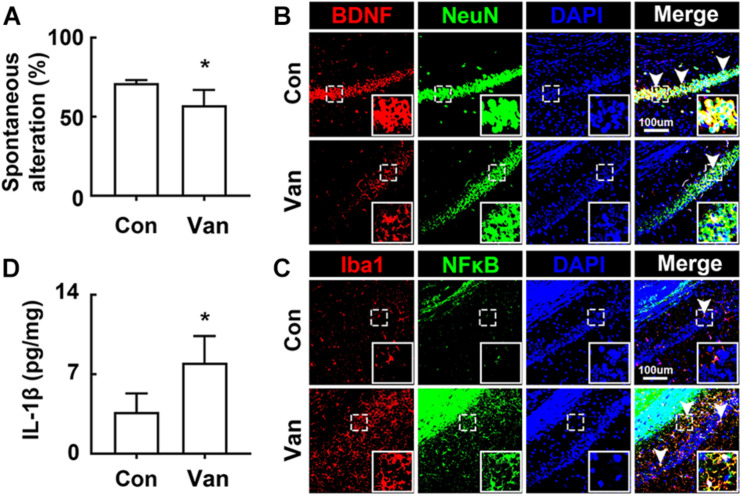
Vancomycin caused the cognitive impairment in mice. **(A)** Effects on the cognitive function, assessed by the Y-maze task. Effects on BDNF^+^/NeuN^+^ **(B)** and NF-κB^+^/Iba1^+^ cell populations **(C)** and IL-1β expression **(D)** in the hippocampus. Con and Van in the *x*-axis of figures indicate groups treated with vehicle and vancomycin (150 mg/kg/day) for 2 days in normal control mice, respectively. All data were expressed as mean ± SD (*n* = 6). *p < 0.05 vs. control group.

Next, we examined the effect of orally administered vancomycin in control mice. Oral gavage of vancomycin significantly caused cognitive impairment in the Y-maze task and increased the IL-1β level in the hippocampus, as previously reported in mice treated with ampicillin (Jang et al., 2018a; Figures 2A,D). Furthermore, vancomycin treatment also increased the BDNF^+^/NeuN^+^ cell population, while the NF-κB^+^/Iba1^+^ cell population decreased (Figures 2B,C).

### Orally Administered Vancomycin and Ampicillin Deteriorated tIsc-Induced Gut Inflammation in Mice

**FIGURE 3:**
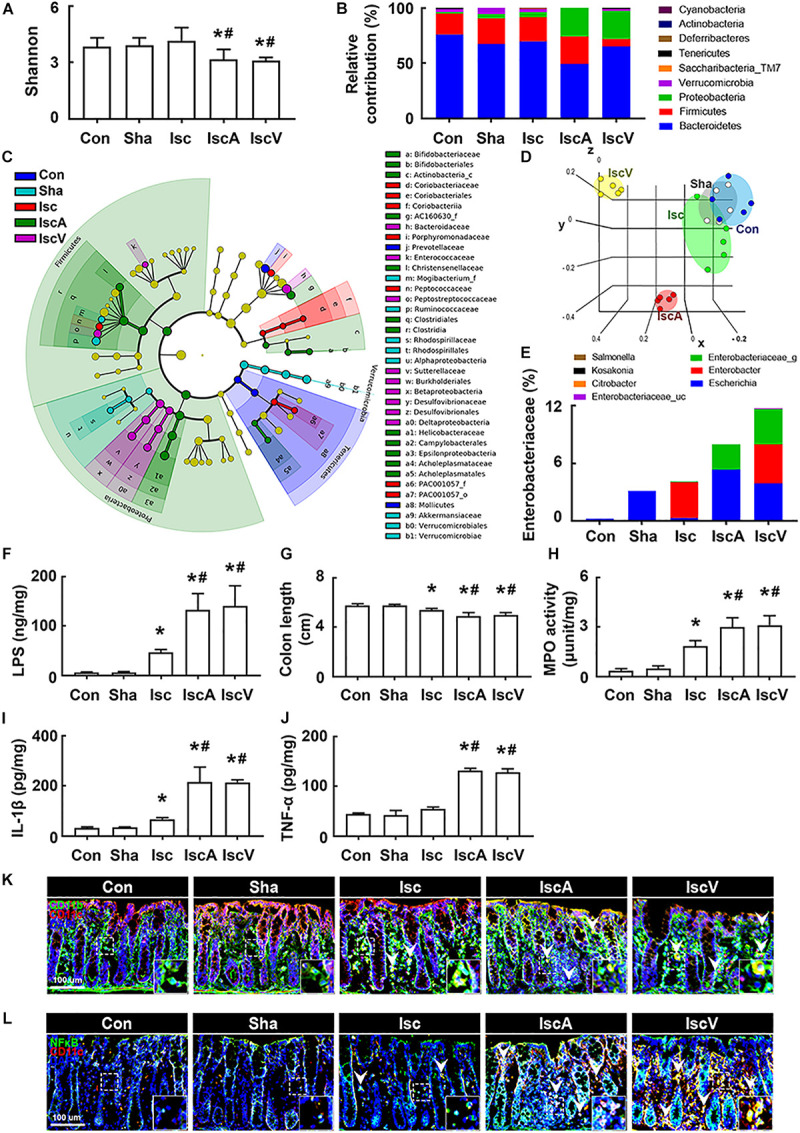
Vancomycin and ampicillin caused the gut dysbiosis and colitis in mice with and without tIsc. Effects on the composition of gut microbiota, analyzed by the pyrosequencing: Shannon **(A)**, phylum **(B)**, cladogram **(C)** generated by LEfSE indicating significant differences in gut microbial abundances among Con (blue), Sha (gray), Isc (green), IscA (red), and IscV (yellow) groups, and principal coordinate analysis (PCoA) plots based on Jensen-Shannon analysis **(D)**. The threshold logarithmic score was set at 2.0 and ranked. Yellow nodes represent species with no significant difference. **(E)** Effects on bacterial population cultured in the DHL agar plate. **(F)** Effects on the fecal LPS level. Effects on colon length **(G)**, myeloperoxidase activity **(H)**, IL-1β **(I)**, and TNF-α expression **(J)** in the colon. Effects on CD11b^+^/CD11c^+^ **(K)** and NF-κB^+^/Iba1^+^ cell **(L)** populations in the colon. Con, Sha, Isc, IscA, and IscV in the *x*-axis of figures indicate groups treated with vehicle in normal control mice, vehicle in sham mice, ampicillin in mice with tIsc, and vancomycin in mice with tIsc, respectively. All data were expressed as mean ± SD (**A–E**, *n* = 5; **F–J**, *n* = 6). *p < 0.05 vs. Sha group. ^#^*p* < 0.05 vs. Isc group.

To understand whether the occurrence of cognitive impairment by antibiotics was associated with gut dysbiosis, we examined the effects of orally administered ampicillin and vancomycin on gut microbiota composition and colitis in mice with tIsc. The induction of tIsc did not significantly affect gut microbiota composition compared to those of control and sham mice. However, oral administration of vancomycin or ampicillin in mice with tIsc significantly reduced α-diversity and shifted β-diversity in the gut microbiota composition (Figures 3A,D). Oral administration of vancomycin significantly increased the Proteobacteria population and decreased the Verrucomicrobia population (Figures 3B,E). Orally administered ampicillin also increased the Proteobacteria population, as previously reported in normal control mice (Jang et al., 2018a). Furthermore, orally administered vancomycin and ampicillin increased the population of Enterobacteriaceae including *Enterbacter* sp. in mice with tIsc (Figure 3C, Supplementary Figure S4A, and Supplementary Tables S1, S2). Therefore, to confirm whether these antibiotic treatments could increase the Proteobacteria population in mice with tIsc, we cultured the fecal bacteria of orally vancomycin-treated mice with tIsc in a DHL agar plate, which is a selective medium for Enterobacteriaceae (Figure 3E). Oral administration of vancomycin significantly increased the population of *Enterobacter xiangfangenesis*, which belongs to the Proteobacteria family, in the DHL agar plate compared to those of control, sham, and tIsc mice. tIsc operation weakly increased fecal LPS levels. Antibiotic treatments increased the fecal LPS level in mice with tIsc more than vehicle treatment (Figure 3F).

tIsc operation itself caused mild colitis in control mice compared to those of control and sham mice (Figures 3G,H). Oral gavage of vancomycin or ampicillin significantly deteriorated tIsc-induced colitis; they dramatically increased colon shortening, myeloperoxidase activity, and TNF-α and IL-1β levels in the colon (Figures 3G–J). Their treatments also increased the CD11b^+^/CD11c^+^ and NF-κB^+^/CD11c^+^ cell (activated macrophage/dendritic cell) populations in the colon (Figures 3K,L).

### Orally Delivered Fecal Transplantations of Vancomycin-Treated tIsc Mice Deteriorated tIsc-Induced Cognitive Impairment and Colitis in Mice

**FIGURE 4:**
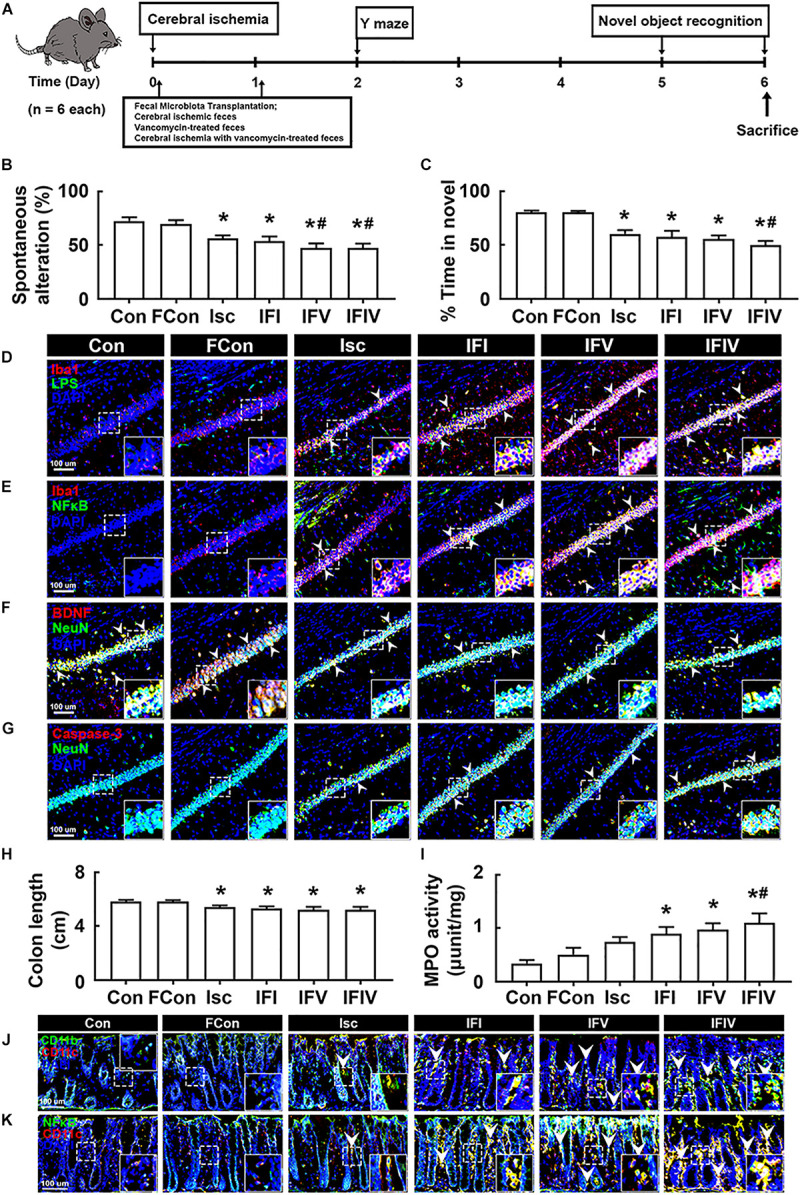
The fecal transplantations of vancomycin-treated mice deteriorated the cognitive impairment and colitis in mice with tIsc. **(A)** Experimental protocol. Effects on the cognitive function, assessed by the Y-maze **(B)** and novel object recognition **(C)**. Effects on LPS^+^/Iba1^+^ **(D)**, NF-κB^+^/Iba1^+^ **(E)**, BDNF^+^/NeuN^+^ **(F)**, and caspase 3^+^/NeuN^+^ cell **(G)** populations in the hippocampus, assessed by a confocal microscope. Effects on colon length **(H)**, myeloperoxidase activity **(I)**, and CD11b^+^/CD11c^+^ **(J)** and NF-κB^+^/Iba1^+^ **(K)** cell populations in the colon. Con, FC, Isc, IFI, IFV, and IFIV in the *x*-axis of figures indicate groups orally treated with vehicle into normal control mice, feces of sham mice into normal control mice, vehicle into mice with tIsc, Isc mouse feces into mice with tIsc, vancomycin-treated normal control mice into tIsc mice, and vancomycin-treated tIsc mouse feces into mice with tIsc, respectively. All data were expressed as mean ± SD (*n* = 6). *p < 0.05 vs. FC group. ^#^*p* < 0.05 vs. Isc group.

To understand whether the induction of cognitive impairment and colitis by antibiotic treatments was associated with gut dysbiosis, we orally transplanted the feces of sham mice into normal control mice (FC), tIsc-induced mice into tIsc mice (IFI), vancomycin-treated normal control mice into tIsc mice (IFV), or vancomycin-treated tIsc mice into mice with tIsc (IFIV) and examined their effects on the occurrence and development of cognitive impairment and colitis (Figure 4A). The occurrence of cognitive impairment by fecal transplantation of IFI into tIsc mice was not significantly different to that in mice with tIsc (Figures 4B,C). However, the fecal transplantation of IFV or IFIV into tIsc mice caused more severe cognitive impairment in Y-maze and novel object recognition tasks compared to those in control or IFI feces-transplanted mice. Orally delivered fecal transplantation of IFIV or IFV also significantly increased the NF-κB^+^/Iba1^+^ and LPS^+^/Iba1^+^ cell populations in the hippocampus (Figures 4D,E). Furthermore, treatment with IFIV or IFV increased the caspase 3^+^/NeuN^+^ (apoptotic neuron) cell population in the hippocampus, while the BDNF^+^/NeuN^+^ cell population was suppressed (Figures 4F,G). Orally delivered fecal transplantation of IFIV or IFV into mice also caused colon shortening and induced myeloperoxidase activity in the colon more severely than those of control and IFI feces-transplanted mice (Figures 4H,I). Treatment with IFIV or IFV also increased the CD11b^+^/CD11c^+^ and NF-κB^+^/CD11c^+^ cell populations in the colon (Figures 4J,K). Overall, the effect of IFV fecal transplantation on the cognitive impairment colitis was not significantly different with that of IFIV fecal transplantation.

### *Enterobacter xiangfangenesis* Caused Cognitive Impairment in Mice With and Without tIsc

**FIGURE 5:**
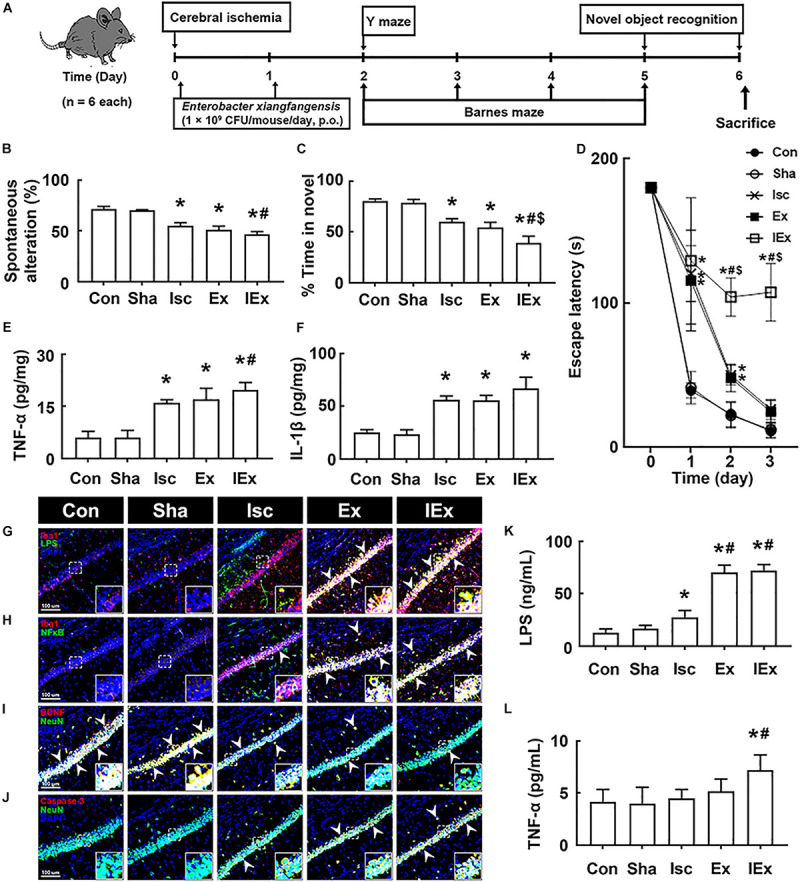
*Enterobacter xiangfangenesis* caused the cognitive impairment in mice with and without tIsc. **(A)** Experimental protocol. Effects on the cognitive function, assessed by the Y-maze **(B)**, novel object recognition **(C)**, and Banes maze tasks **(D)**. Effects on TNF-α **(E)** and IL-1β expression **(F)** in the hippocampus, assessed by ELISA. Effects on LPS^+^/Iba1^+^ **(G)**, NF-κB^+^/Iba1^+^ **(H)**, BDNF^+^/NeuN^+^ **(I)**, and caspase 3^+^/NeuN^+^ cell **(J)** populations in the hippocampus, assessed by a confocal microscope. Effects on LPS **(K)** and TNF-α **(L)** levels in the blood. Con, Sha, Isc, Ex, and IEx in the *x*-axis of figures indicate groups treated with vehicle in normal control mice, vehicle in sham mice, vehicle in mice with tIsc, *Enterobacter xiangfangenesis* in control mice, and *Enterobacter xiangfangenesis* in mice with tIsc, respectively. All data were expressed as mean ± SD (*n* = 6). *p < 0.05 vs. Sha group. ^#^*p* < 0.05 vs. Isc group. ^$^*p* < 0.05 vs. Ex group.

Oral administration of vancomycin significantly increased the *Enterobacter xiangfangenesis* population in mice with tIsc. Therefore, to understand whether *Enterobacter xiangfangenesis* was associated with the occurrence and development of cognitive impairment, we examined its effect on cognitive impairment in mice with and without tIsc in Y-maze, novel object recognition, and Banes maze tasks (Figure 5A). Oral gavage of *Enterobacter xiangfangenesis* severely deteriorated the cognitive impairment in mice with or without tIsc compared to those in control and sham mice (Figures 5B–D). *Enterobacter xiangfangenesis* treatment deteriorated cognitive impairment in mice with tIsc more severely than in mice without tIsc. *Enterobacter xiangfangenesis* treatment also significantly increased TNF-α and IL-1β expression in the hippocampus (Figures 5E,F). Exposure of mice with tIsc to *Enterobacter xiangfangenesis* significantly decreased the BDNF^+^/NeuN^+^ neuron cell population in the hippocampus, while the caspase 3^+^/NeuN^+^ (apoptotic neuron) cell and activated microglia (LPS^+^/Iba1^+^ and NF-κB^+^/Iba1^+^ cells) populations increased (Figures 5G–J). Furthermore, treatment with *Enterobacter xiangfangenesis* increased LPS and TNF-α levels in the blood of mice with or without tIsc (Figures 5K,L).

### *Orally Gavaged Enterobacter xiangfangenesis* Caused Colitis and Gut Microbiota Alteration in Mice With and Without tIsc

**FIGURE 6:**
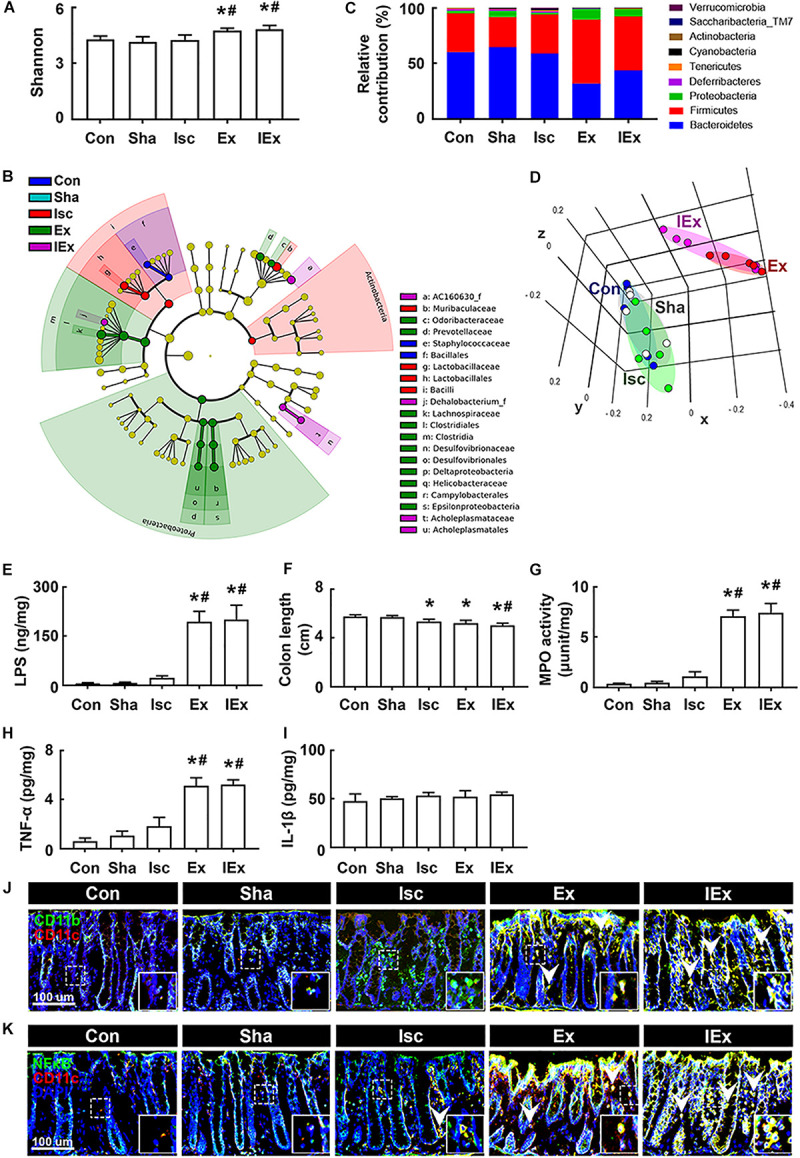
*Enterobacter xiangfangenesis* caused the gut dysbiosis and colitis in mice with and without tIsc. Effects on the composition of gut microbiota, analyzed by the pyrosequencing: Shannon **(A)**, cladogram **(B)** generated by LEfSE indicating significant differences in gut microbial abundances among Con (blue), Sha (gray), Isc (green), Ex (red), and IEx (purple) groups, phylum **(C)**, and principal coordinate analysis (PCoA) plots based on Jensen-Shannon analysis **(D)**. The threshold logarithmic score was set at 2.0 and ranked in cladogram. Yellow nodes represent species with no significant difference. **(E)** Effects on the fecal LPS level. Effects on colon length **(F)**, myeloperoxidase activity **(G)**, and TNF-α **(H)** and IL-1β **(I)** expression in the colon. Effects on CD11b^+^/CD11c^+^ **(J)** and NF-κB^+^/Iba1^+^ cell **(K)** populations in the colon. Con, Sha, Isc, Ex, and IEx in the *x*-axis of figures indicate groups treated with vehicle in normal control mice, vehicle in sham mice, vehicle in mice with tIsc, *Enterobacter xiangfangenesis* in control mice, and *Enterobacter xiangfangenesis* in mice with tIsc, respectively. All data were expressed as mean ± SD (**A–D**, *n* = 5; **E–I**, *n* = 6). *p < 0.05 vs. Sha group. ^#^*p* < 0.05 vs. Isc group.

Next, we examined the effects of orally gavaged *Enterobacter xiangfangenesis* on gut microbiota composition and colitis in mice. The induction of tIsc in mice did not significantly shift the gut microbiota composition compared to that of *Enterobacter xiangfangenesis-*treated mice (Figures 6A–D, Supplementary Figure S4B and Supplementary Tables S3, S4). The exposure of mice with or without tIsc to *Enterobacter xiangfangenesis* fluctuated α-diversity and shifted β-diversity in the gut microbiota composition. Furthermore, treatment with *Enterobacter xiangfangenesis* increased the Firmicutes and Proteobacteria populations, while the Bacteroidetes population decreased. These treatments also increased the fecal LPS levels in mice with or without tIsc (Figure 6E).

Furthermore, exposure to *Enterobacter xiangfangenesis* induced colon shortening and myeloperoxidase activity in mice with and without tIsc (Figures 6F,G). The difference in *Enterobacter xiangfangenesis-*induced colon shortening and myeloperoxidase activity between mice with and without tIsc was not significant. Treatment with *Enterobacter xiangfangenesis* also upregulated TNF-α expression in mice with and without tIsc (Figure 6H). However, the IL-1β level was not affected (Figure 6I). Treatment with *Enterobacter xiangfangenesis* also increased the NF-κB^+^/CD11c^+^ and CD11b^+^/CD11c^+^ cell populations in the colon (Figures 6J,K). *Enterobacter xiangfangenesis* treatment significantly deteriorated colitis in mice with tIsc.

## Discussion

Recently, a series of studies have highlighted that gut microbiota bidirectionally communicates with the brain and gut dysbiosis is closely associated with the occurrence of psychiatric disorders, including Alzheimer’s disease (AD) (Bercik et al., 2011; Quigley, 2017; Rieder et al., 2017). The exposure of humans and animals to antibiotics, particularly oral administration, reduces bacterial diversity and induces antibiotic resistance, gut dysbiosis, and psychiatric disorders depending on the route of their administration: orally administered antibiotics such as ampicillin generally altered gut microbiota more severely than parentally injected ones (Zhang et al., 2013; Modi et al., 2014; Jang et al., 2018a; Zhou et al., 2020). Fröhlich et al. (2016) reported that oral gavage of an ampicillin, vancomycin, neomycin, bacitracin, and meropenem cocktail, which did not enter the brain, disrupted gut microbiota and impaired cognitive function in a novel object recognition task. Oral administrations of other antibiotic mixtures, such as a cocktail of ampicillin, vancomycin, metronidazole, neomycin, and amphotericin-B, also modified gut microbiota composition, caused anxiety, and/or impaired cognitive function in mice (Desbonnet et al., 2015; Hoban et al., 2016). Oral administration of ampicillin, which exhibits the same spectrum as amoxicillin *in vitro*, significantly caused colitis and anxiety in mice despite short-term treatment (Becattini et al., 2016; Jang et al., 2018a). However, oral administration of early life antibiotic penicillin decreased anxiety-like behavior in male mice (Leclercq et al., 2017). Oral administration of antibiotics (a cocktail of neomycin, bacitracin, and pimaricin) reduced anxiety in mice (Bercik and Collins, 2014). Nevertheless, to reduce the risk of pathogen infections, antibiotics are recommended in patients with ischemic brain injury (Xi et al., 2017). Therefore, to understand this discrepancy, the effects of the oral administration of vancomycin and ampicillin, which are recommended in patients with ischemic brain injury, on the occurrence of psychiatric disorders including cognitive impairment should be elucidated.

In the present study, we found that the oral administration of vancomycin or ampicillin, despite short-term treatment, markedly increased the fecal Proteobacteria population and LPS production in tIsc mice, and in normal control mice orally gavaged with ampicillin (Jang et al., 2018c). Jang et al. (2018c) also reported that oral administration of ampicillin significantly increased the Proteobacteria population and bacterial LPS production in mice. These data are consistent with previous reports that oral ampicillin (Zhang et al., 2013) and vancomycin (Isaac et al., 2017) severely disrupted the gut microbiota of healthy control mice. These results suggest that the oral administration of vancomycin and ampicillin can cause gut dysbiosis, whether in healthy or impaired conditions, and the application of these antibiotics by oral administration for the therapy of brain ischemia can severely increase gut microbiota LPS production with gut dysbiosis. We also found that these antibiotic treatments deteriorated colitis in mice with tIsc; they induced myeloperoxidase activity, active macrophages/dendritic cells (CD11b^+^/CD11c^+^ and NF-κB^+^/CD11c^+^ cells) population, and suppressed gut tight junction protein expression in the colon. Furthermore, antibiotic treatment increased the blood LPS level and gut Proteobacteria population in tIsc mice more than in tIsc mice not treated with antibiotics. Jang et al. (2018a) reported that ampicillin treatment increased the blood LPS levels, gut Proteobacteria population, and absorption of orally gavaged fluorescein-conjugated dextran from the gut membrane into the blood in mice. These results suggest that oral administration of vancomycin and ampicillin can induce gut Proteobacteria population and bacterial LPS production and accelerate the absorption of fecal LPS into the blood by inducing gut dysbiosis-induced colitis (leaky gut) and suppressing gut tight junction protein expression. Furthermore, the induction of gut dysbiosis by orally administered antibiotics, such as the induction of the Proteobacteria population by ampicillin or vancomycin, may elevate the translocation of LPS into the brain, particularly the hippocampus, resulting in neuroinflammation, as previously reported (Banks et al., 2015). Moreover, oral administration of antibiotics (vancomycin and ampicillin) increased activated microglial (LPS^+^/Iba1^+^ and NF-κB^+^/Iba1^+^) cell populations, induced increased NF-κB activation, and suppressed the activated neuron (BDNF^+^/NeuN^+^) cell population in the hippocampus of tIsc mice. In addition, vancomycin treatment caused cognitive impairment in normal control mice. Jang et al. (2018a) reported that ampicillin treatment can cause psychiatric disorders and colitis in control mice. We also found that the number of neuron cells suppressed by antibiotics was inversely proportional to the number of NF-κB^+^/Iba1^+^ cells in the hippocampus with tIsc and NF-κB^+^/CD11c^+^ cells in the colon. The activated neuron (BDNF^+^/NeuN^+^) cell population was in inverse proportion to the activated microglial (LPS^+^/Iba1^+^ and NF-κB^+^/Iba1^+^) cell populations in the hippocampus. The fecal LPS level was inversely proportional to tight junction protein expression. LPS recognition proteins such as toll-like receptor 4 are increased in the brains of patients with neuro-inflammation (Buchanan et al., 2010; Li et al., 2016). These results suggest that oral administration of vancomycin or ampicillin can increase the absorption of gut microbiota products including LPS through the overgrowth of the Proteobacteria population in the gut into the blood and brain, which can accelerate hippocampal inflammation, resulting in cognitive impairment.

While these data illustrated the profound impact of oral antibiotics on gut microbiota disruption and associated host responses, previous reports illustrated that changing ampicillin and vancomycin from oral to injective administration significantly alleviated the damages on gut microbiota (Zhang et al., 2013; Isaac et al., 2017; Zhou et al., 2020). These findings are consistent with the report by Vogt et al. (2016), showing that intraperitoneally injected minocycline does not display anxiolytic or anti-depressant behaviors in mice. Thus it is plausible that changing vancomycin and ampicillin administration from oral to injection may help mitigate the reported detrimental impact of the antibiotic treatment in tIsc hosts.

The fecal transplantations of vancomycin-treated mice severely deteriorated colitis in the transplanted tIsc mice compared to those in mice transplanted with mouse feces not treated with vancomycin; they induced colon shortening, myeloperoxidase activity, and infiltration of activated macrophages and dendritic cells (CD11b^+^/CD11c^+^ and NF-κB^+^/CD11c^+^) into the colon. The transplantation of vancomycin-treated mouse feces, which contained a higher abundance of gut Proteobacteria than those of vancomycin-untreated mouse feces, also caused cognitive impairment and hippocampal inflammation compared to those in IFI feces-transplanted mice; it impaired the cognitive function and induced the hippocampal NF-κB-activated microglia cell population. However, the transplantation of control or tIsc mouse feces into tIsc mice did not deteriorate colitis and cognitive impairment. Furthermore, we found that treatment with the antibiotics vancomycin and ampicillin disrupted gut microbiota composition in mice with and without tIsc; they significantly increased the population of Proteobacteria including *Enterobacter xiangfangenesis*. Treatment with vancomycin or ampicillin more severely caused gut dysbiosis in mice with tIsc than in mice without tIsc. Thus, they induced colon shortening and myeloperoxidase activity, activated macrophage and dendritic cell (CD11b^+^/CD11c^+^ and NF-κB^+^/CD11c^+^) populations in the colon, impaired cognitive function, and reduced the BDNF^+^/NeuN^+^ cell population in the hippocampus and increased the activated microglia (LPS^+^/Iba1^+^ and NF-κB^+^/Iba1^+^ cells) population. Atli et al. (2006) reported that orally administered amoxicillin caused adverse neurological side effects in children such as anxiety, hyperactivity, confusion, and behavioral changes. Jang et al. (2018a) also reported that oral administration of ampicillin or ampicillin-inducible *Klebsiella oxytoca* in the gut microbiota of mice caused gut microbiota disruption, including an increase in the Preteobacteria population, and hippocampal inflammation in mice. These results suggest that oral administration of the antibiotics vancomycin and ampicillin for the therapy of patients with brain ischemia can stimulate to facilitate the proliferation of the Proteobacteria population, such as *Enterobacter xiangfangenesis*, which can cause gut and hippocampal inflammation and then accelerate the progression of cognitive decline. Antibiotics-inducible gut dysbiosis can affect the secretion of neuroactive, endocrinal, and immunological molecules in the intestine by inducing NF-κB activation by gut bacteria byproducts such as LPS, resulting in the deterioration of cognitive impairment progression.

**FIGURE 7:**
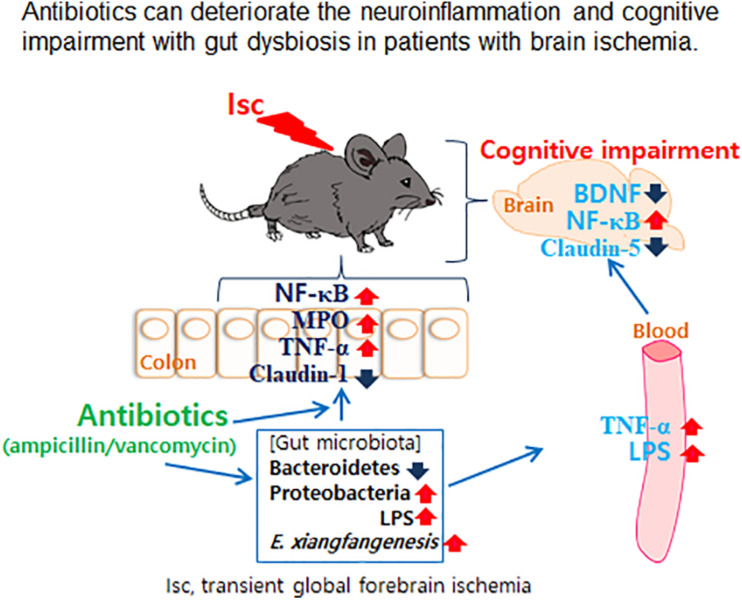
Proposed mechanism of cognitive impairment with gut dysbiosis by antibiotics.

In conclusion, treatment with the antibiotics vancomycin and ampicillin by oral administration deteriorated the cognitive impairment with gut dysbiosis in both healthy and tlsc mice (Figure 7). The finding has direct implications for clinic application of oral vancomycin and ampicillin in patients with brain ischemia. While it is recognized that changing antibiotic administration from oral to injection alleviated gut microbiota disruption, its applicability in clinical treatment for patients with brain ischemia needs further validation.

## Materials and Methods

### Culture of Gut Bacteria

For the culture of gut bacteria, the fresh feces of mice (approximately 0.1 g) was suspended in a ninefold volume of general anaerobic medium (GAM) broth (Nissui Pharmaceutical Co., Tokyo, Japan), diluted in a stepwise manner, and inoculated directly onto selective agar plates (Jang et al., 2018b). Deoxycholate and hydrogen sulfide lactose (DHL) agar (Nissui Pharmaceutical Co.) plates were aerobically cultured for 1 day at 37°C and blood liver (BL) agar (Nissui Pharmaceutical Co.) plates were anaerobically cultured for 3 days at 37°C.

*Enterobacter xiangfangenesis* was cultured in GAM broth. Briefly, isolated bacteria were anaerobically cultured in 0.3 L of GAM broth at 37°C (an optical density at 600 nm, 0.6–0.8), centrifuged at 5,000 *g* for 20 min, and washed with saline twice. Collected cells (5 × 10^9^ colony forming unit [CFU]/mL) were suspended in 1% glucose (for *in vivo* study). Isolated bacteria were identified by Gram staining, a sugar utilization test, and 16S rRNA sequencing.

### Animals

Male C57BL/6 mice (6 weeks old, 20–22 g) were obtained from the Orient Animal Breeding Center (Seoul, South Korea) and housed in controlled standard conditions. All mice were housed in plastic cages with a raised wire floor (3 mice per cage) at 20–22°C, 50 ± 10% humidity, and 12-h light/dark cycle (lights on from 07:30 to 19:30). They were fed standard laboratory chow and water *ad libitum*. Mice were used in the experiments after the acclimation for 1 week.

### Preparation of Mice With tIsc and Treatment With Antibiotics

Mice with tIsc were prepared according to the method of Lee et al. (2016). Each group consisted of eight mice. Briefly, their bilateral common carotid arteries were occluded for 15 min. Mice were anesthetized with 2% isoflurane and maintained with 1% isoflurane maintenance during ischemia-induced surgery. In sham mice, the bilateral common carotid arteries were not occluded and only isolated from the adjacent vag us nerve. The tIsc- and sham-operated mice did not die during the experiment. Ampicillin (200 mg/kg/day) and vancomycin (150 mg/kg/day), which were converted from mouse doses to human equivalent doses (ampicillin, 1–2 g/60 kg/day; vancomycin, 0.5–2 g/60 kg/day) based on body surface area [animal dose (mg/kg) × 3/37] (Nair and Jacob, 2016), were orally gavaged daily for 2 days from 24 h after the occlusion.

To examine the effects of gut bacteria on the occurrence and development of the cognitive impairment in patients with brain ischemia, the suspension of mouse feces (2.5 mg/mouse/day) treated with or without vancomycin or *Enterobacter xiangfangenesis* (1 × 10^9^ CFU/mouse/day) were orally gavaged once a day for 5 days in mice. Each group consisted of 6 mice. Animal experiments were conducted with two replicates. To exclude the effects of orally administered antibiotics in the feces of mice, fecal suspension was prepared as follows: fresh feces were collected on the 5th day after the final vancomycin treatment, suspended in GAM broth on ice, centrifuged at 2,000 *g* for 15 min at 4°C, washed with 1% glucose, and used as the fecal microbial suspension (25 mg/mL suspended in 1% glucose).

Memory-related behaviors were measured using a Y-maze, novel object recognition, and a Banes maze on the 5th day after treatment with fecal suspension or bacterial suspension. At the end of the behavioral test, all animals were sacrificed by CO_2_ inhalation. Blood, brains, and colons were collected. The brain and colon were stored at –80°C for the assay of biochemical markers. For the immunohistochemistry assay, mice were transcardially perfused with 4% paraformaldehyde for brain and colon tissue fixation. Brains and colon tissues were post-fixed with 4% paraformaldehyde for 4 h, cytoprotected in 30% sucrose solution, frozen, and cut using a cryostat (Leica, Nussloch, Germany) (Kim et al., 2020).

### Behavioral Tasks

The Y-maze task was carried out in a three-arm horizontal maze (40 cm long and 3 cm wide with 12-cm-high walls) (Jang et al., 2018b). A mouse was initially placed within one arm and the sequence of arm entries was recorded for 8 min. A spontaneous alternation was defined as sequential entries into three arms such as ABC, BCA, and CAB and was calculated as the ratio (%) of spontaneous to possible alternations. A novel object recognition task was performed in the apparatus consisting of a dark-open field box (45 × 45 × 50 cm), as previously reported (Lee et al., 2018). Briefly, in the first trial, a mouse was placed in the box containing two identical objects and the frequency of touching each object was recorded for 10 min. In the second trial conducted 24 h after the first trial, a mouse was placed in the box containing one of the old objects used in the first trial and a new object. Novel object recognition was calculated as the ratio of the number of times touching the new object to the sum of the touching frequencies. A Barnes maze task was performed in the apparatus consisting of a circular platform (89 cm in diameter) with 20 holes (5 cm in diameter) situated evenly around the perimeter and an escape box located under only one of the holes below the platform, as previously reported (Kim et al., 2020). Briefly, the training/acquisition phase finished after the mouse entered the escape box or after a maximum test duration (5 min), following which the mouse was allowed to stay in the escape box for 30 s and then moved to the cage. If the mouse failed to find the escape box within 5 min, it was led to the escape box. The task was performed for 5 consecutive days.

### Immunofluorescence Assay

Immunostaining was performed, as previously reported (Jang et al., 2018b,c). Briefly, brain and colon tissue sections were washed with PBS, blocked with normal serum, incubated with antibodies for Iba1 (1:200, Abcam), LPS (1:200, Millipore), NF-κB (1:100, Cell Signaling), CD11b (1:200, Abcam), CD11c (1:200, Abcam), and/or NeuN (1:200, Millipore) antibodies overnight, and treated with the secondary antibodies for 2 h. Secondary antibodies conjugated with Alexa Fluor 488 (1:200, Invitrogen) or Alexa Fluor 594 (1:200, Invitrogen) were then treated to enable visualization. Nuclei were stained with 4’,6-diamidino-2-phenylindole, dilactate (DAPI, Sigma). Immunostained samples were scanned with a confocal laser microscope.

### Enzyme-Linked Immunosorbent Assay (ELISA)

Brain and colon tissues were removed 2 h after the performance of the final task and homogenized with RIPA lysis buffer with a 1% protease inhibitor cocktail and a phosphatase inhibitor cocktail on ice (Jang et al., 2018a). The lysates were centrifuged at 10,000 *g* and 4°C for 10 min. For ELISA, the capture antibody for each cytokine was coated in a 96-well plate according to the manufacturer’s protocol (eBioscience, San Diego, CA) and the resulting supernatants were transferred into the 96-wells. Thereafter the detection antibody solution was treated and measured the absorbance at 410 nm (Jang et al., 2018b).

### Myeloperoxidase Activity and Limulus Amebocyte Lysate (LAL) Assays

Myeloperoxidase activity was measured, as previously reported (Jang et al., 2018a). Fecal and blood endotoxin contents were determined using the diazo-coupled LAL assay kit (Cape Cod Inc., E. Falmouth, MA), as previously reported (Kim et al., 2012).

### Illumina iSeq Sequencing

Genomic DNA was extracted from the fresh stool of five mice (not transcardially perfused with 4% paraformaldehyde for brain and colon tissue sections) using a commercial DNA isolation kit (QIAamp DNA stool mini kit), as previously reported (Kim et al., 2020). The extracted genomic DNA was amplified using barcoded primers, which targeted the V4 region of the bacterial 16S rRNA gene. The sequencing for equimolar concentration of each amplicon was performed using Illumina iSeq 100 (San Diego, CA) (Kim et al., 2020). Reads taken from different samples were classified by unique barcodes of each polymerase chain reaction product and the target region in barcoded primers was identified. All of the linked sequences including adapter, barcode, and linker and low quality sequences (reads with two or more indefinite nucleotides, a low quality score, or < 500 bp) were eliminated. Potential chimeric sequences were confirmed by the Bellerophon formula. The taxonomic sorting of each read was assigned against the EzTaxon-e database^1^, which has the 16S rRNA gene sequence of type strains that have valid published names and representative species level phylotypes of either cultured or uncultured entries in the GenBank database with complete hierarchical taxonomic classification from phyla to species. The 16S rRNA gene sequences originating from our study were deposited in NCBI’s SRA (SRX4051778∼4051782, SRX3153173, SRX3153178∼3153180, SRX3153182). The species richness of samples was determined using the CLcommunity program. Subsampling was randomly performed to equalize the read size of tested samples to compare the different read size within tested samples. For the comparison of the estimated operational taxonomic units (OTUs) between tested samples, shared OTUs were obtained with the XOR analysis of the CLcommunity program. Pyrosequencing reads were deposited in the NCBI’s short read archive under accession number PRJNA507690.

### Ethics Statement

All experimental protocols were approved by the Institutional Animal Care and Use Committee of the Kyung Hee University (IACUC Number: KUASP(SE)-18033) and performed according to the University Guide for Laboratory Animals Care and Usage.

### Statistical Analysis

All experimental data are indicated as mean ± standard deviation (SD) (Supplementary Table S1) and conducted by Graph-Pad Prism 8 (GraphPad Software Inc., San Diego, CA). The significance for data was analyzed using a non-parametric Mann Whitney test and one-way analysis of variance with *post hoc* Bonferroni’s or Holm Sidak’s multiple comparison test (*p* < 0.05). *p*-values in the present experiments are indicated in Supplementary Table S2.

## Data Availability Statement

The datasets presented in this study can be found in online repositories. The names of the repository/repositories and accession number(s) can be found in the article/Supplementary Material.

## Ethics Statement

The animal study was reviewed and approved by the Institutional Animal Care and Use Committee of the Kyung Hee University.

## Author Contributions

K-EL and D-HK: conceptualization, writing – original draft, and writing – review and editing. K-EL and J-KK: experiment and data analysis. All authors contributed to the article and approved the submitted version.

## Conflict of Interest

The authors declare that the research was conducted in the absence of any commercial or financial relationships that could be construed as a potential conflict of interest.

## Footnotes

1 http://eztaxon-e.ezbiocloud.net

## Supplementary Material

Supplementary Figure 1 Average number of error hole visits during the Barnes maze test in Figure 1D.

Supplementary Figure 2 Oral administration of vancomycin or ampicillin increased Iba1 + cell population in the hippocampus.

Supplementary Figure 3 Oral gavage of vancomycin or ampicillin increased NF-κB^+^/Iba1^+^ **(A)**, LPS^+^/Iba1^+^ **(B)**, and caspase 3^+^/NeuN^+^ cell **(C)** populations and reduced BDNF^+^/NeuN^+^ cell population **(D)** in the hippocampus, assessed by a confocal microscope.

Supplementary Figure 4 Antibiotics **(A)** and *Enterobacter xiangfangenesis* **(B)** caused gut alteration (LDA score) in mice.

Supplementary Table 1 The gut microbiota composition ratio at the family level in ischemia-induced mice treated with or without antibiotics.

Supplementary Table 2 The gut microbiota composition ratio at the genus level in ischemia-induced mice treated with or without antibiotics.

Supplementary Table 3 The gut microbiota composition ratio at the family level in ischemia-induced mice treated with or without *Enterobacter xiangfangenesis.*

Supplementary Table 4 The gut microbiota composition ratio at the genus level in ischemia-induced mice treated with or without *Enterobacter xiangfangenesis.*

Supplementary Table 5 Means and SD of raw data in the experiments.

Supplementary Table 6 *P*-values in the experiments.
