# Supplementary material for: Orally Administered Antibiotics Vancomycin and Ampicillin Cause Cognitive Impairment With Gut Dysbiosis in Mice With Transient Global Forebrain Ischemia
Source: Front Microbiol. 2020 Nov 26;11:564271. doi: 10.3389/fmicb.2020.564271 (PMC7726352; doi:10.3389/fmicb.2020.564271)
Supplement: Supplementary Figure 1 — Average number of error hole visits during the Barnes maze test in Figure 1D. [file Data_Sheet_1.docx]

**[Supporting information]**

**Antibiotics cause cognitive impairment with gut dysbiosis in mice with the transient global forebrain ischemia**


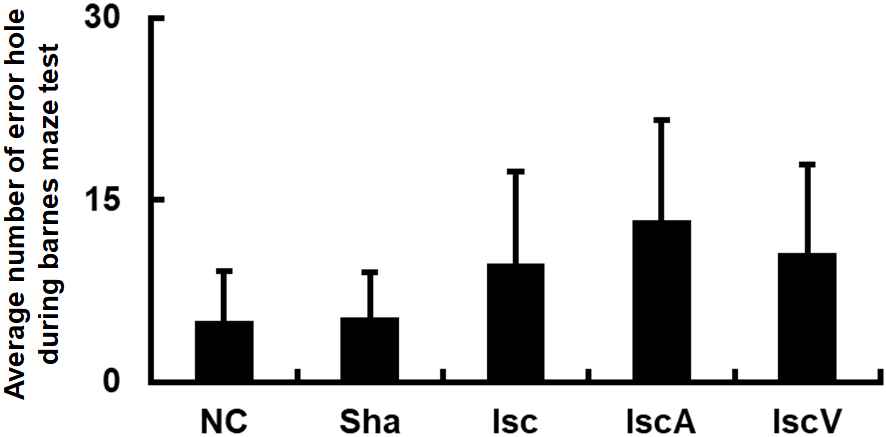


**Supplement Figure S1**. Average number of error hole visiting during Barnes maze test in Figure 1D.


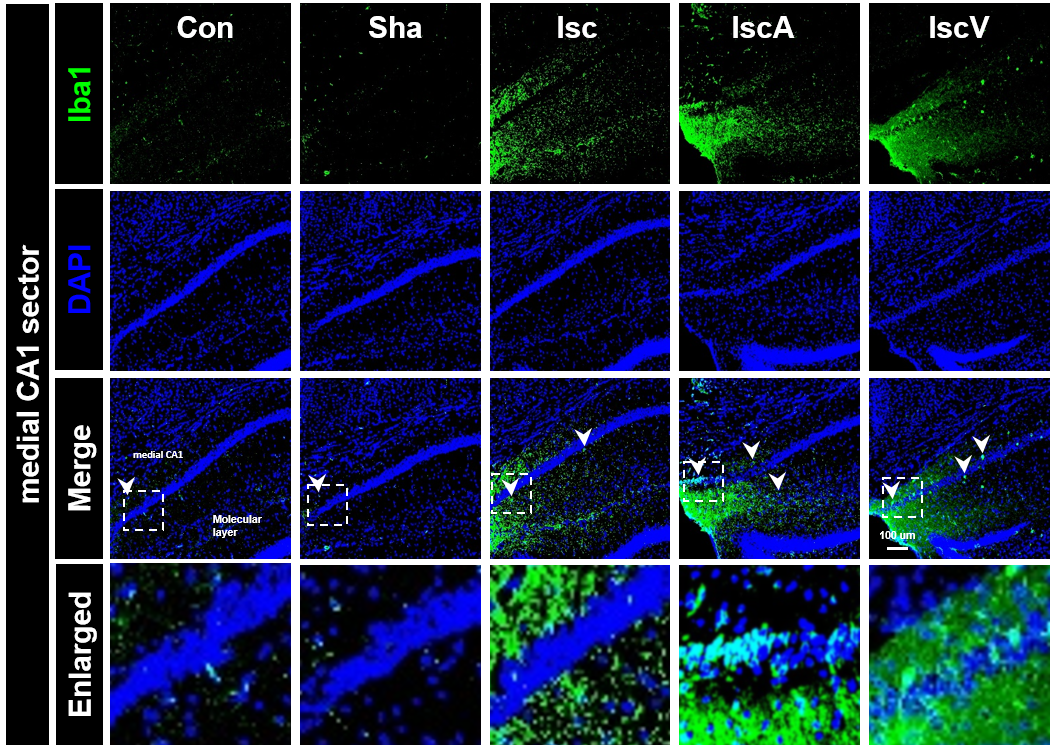

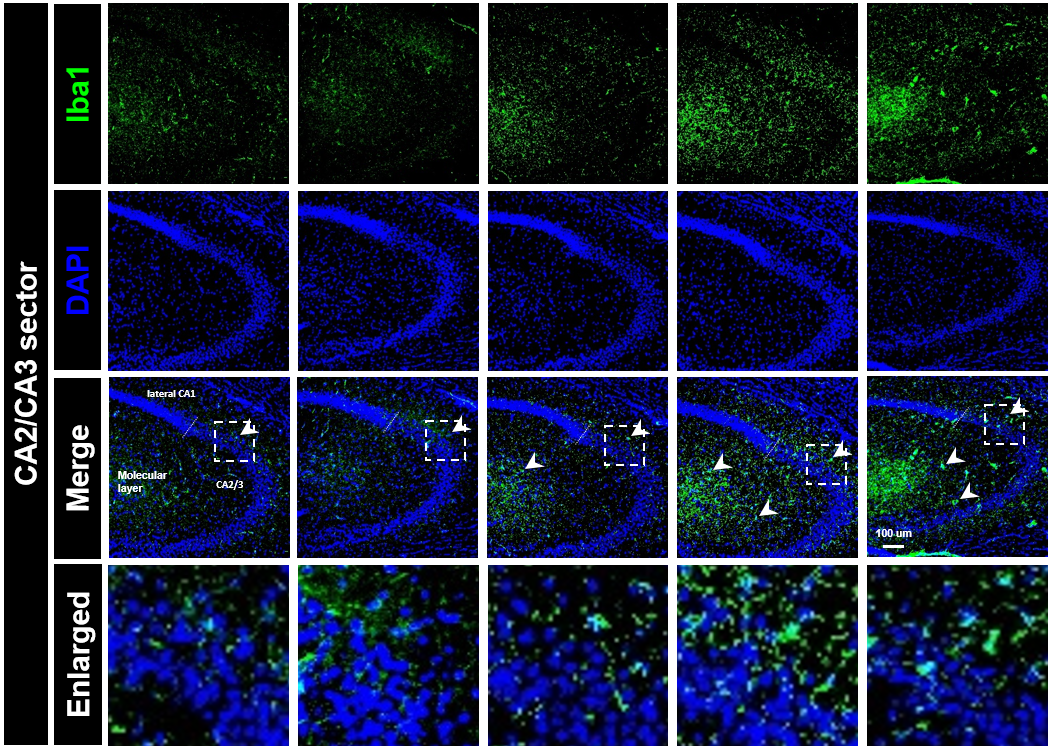


**Supplement Figure S2**. Oral administration of vancomycin or ampicillin increased Iba1^+^ cell population in the hippocampus. Con, Sha, Isc, IscA, and IscV in the x-axis of figures indicate groups treated with vehicle in normal control mice, vehicle in sham mice, vehicle in mice with Isc, ampicillin in mice with Isc, and vancomycin in mice with Isc, respectively.

(A) (B)


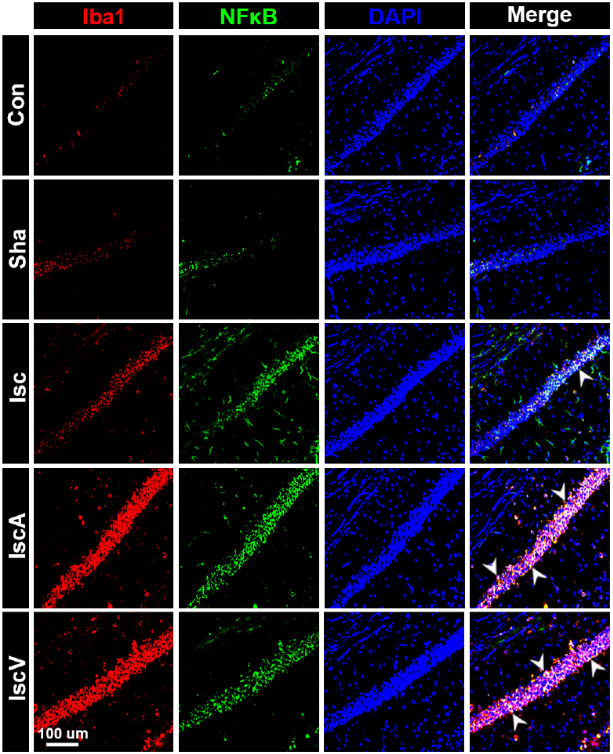

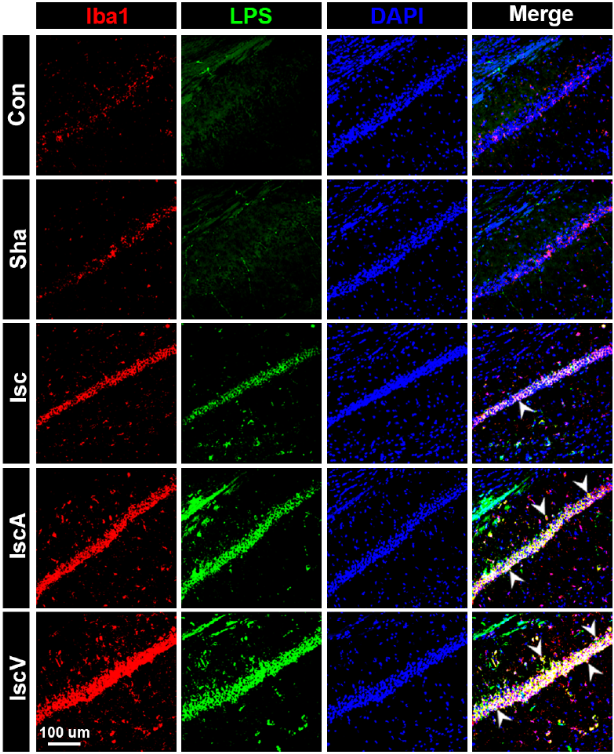


(C) (D)


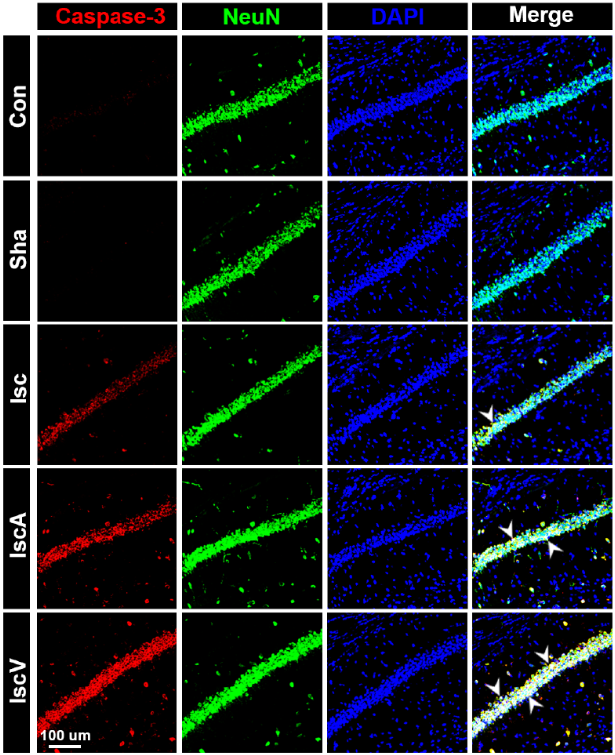

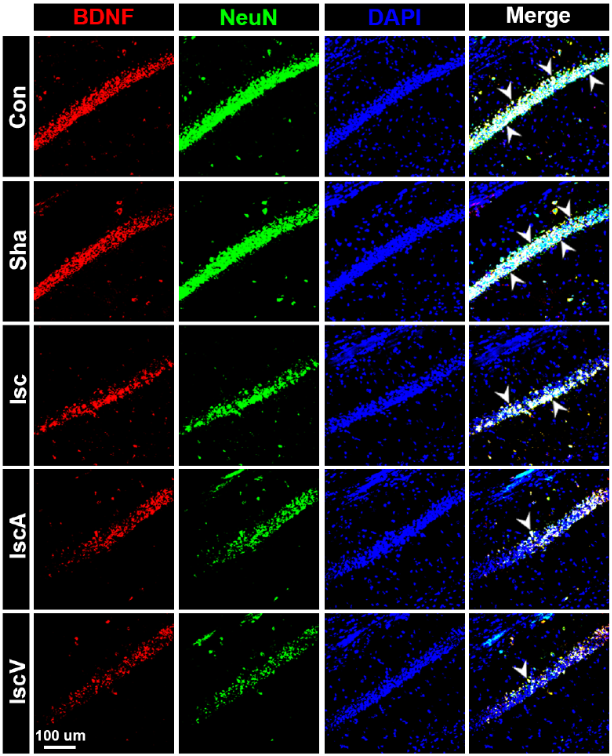


**Supplement Figure S3**. Oral gavage of vancomycin or ampicillin increased NF-κB^+^/Iba1^+^ (A), LPS^+^/Iba1^+^ (B), and caspase 3^+^/NeuN^+^ cell (C) populations and reduced BDNF^+^/NeuN^+^ cell population (D) in the hippocampus, assessed by a confocal microscope. Con, Sha, Isc, IscA, and IscV in the x-axis of figures indicate groups orally gavaged with vehicle in normal control mice, vehicle in sham mice, vehicle in mice with Isc, ampicillin in mice with Isc, and vancomycin in mice with Isc, respectively.

(A) (B)


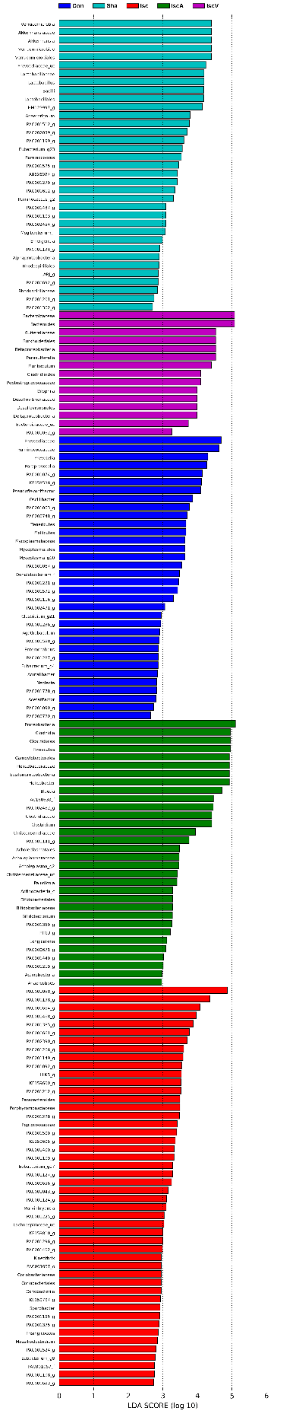

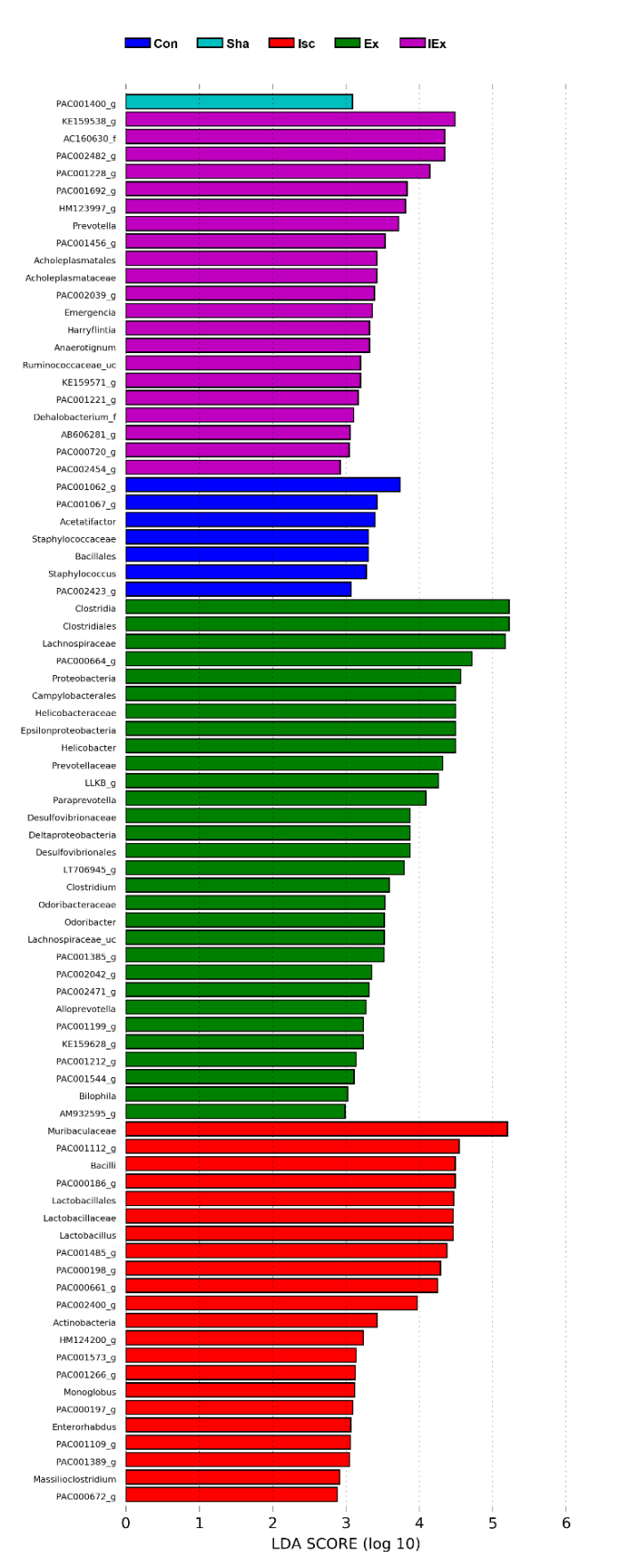


**Supplement Figure S4**. Antibiotics (A) and *Enterobacter xiangfangenesis* (B) caused gut alteration (LDA score) in mice. The described strains were analyzed to the Linear Discriminant Analysis (LDA) along with effect size measurement (LEfSE) in Galaxy (http://huttenhower.sph.harvard.edu/galaxy/). It was used to discriminate significant differentially strains at each taxon level. The threshold logarithmic score set at 2.0 and ranked. Bacterial strains were described based on 16SrRNA sequencing data.

**Supplement Table S1.** The gut microbiota composition ratio at the family level in ischemia-induced mice treated with or without antibiotics

|  | **Average** | | | | | **Standard Deviation** | | | | |
| --- | --- | --- | --- | --- | --- | --- | --- | --- | --- | --- |
| **Taxon Name** | **Con** | **Sha** | **Isc** | **IscA** | **IscV** | **Con** | **Sha** | **Isc** | **IscA** | **IscV** |
| Muribaculaceae | 45.51 | 41.25 | 53.70 | 35.53 | 31.78^#^ | 13.94 | 14.71 | 20.76 | 5.79 | 2.17 |
| Bacteroidaceae | 19.50 | 17.07 | 8.33 | 6.45 | 31.86^#^ | 13.80 | 9.19 | 11.60 | 5.29 | 17.47 |
| Lachnospiraceae | 7.89 | 8.58 | 13.65 | 15.48 | 4.04^#^ | 5.06 | 3.37 | 7.62 | 12.62 | 2.93 |
| Prevotellaceae | 9.34 | 7.13 | 3.71 | 0.03*^,#^ | 0.04^#^ | 3.80 | 4.87 | 2.52 | 0.04 | 0.02 |
| Ruminococcaceae | 7.99 | 8.31 | 5.89 | 1.76*^,#^ | 0.09*^,#^ | 5.36 | 1.36 | 2.42 | 0.96 | 0.03 |
| Akkermansiaceae | 3.05 | 4.76 | 3.23 | 0.01* | 2.04 | 3.16 | 4.49 | 4.45 | 0.01 | 1.20 |
| Lactobacillaceae | 0.66 | 3.01 | 0.43 | 0.14 | 0.35 | 0.41 | 5.12 | 0.42 | 0.06 | 0.11 |
| Clostridiaceae | 0.59 | 1.07 | 0.05 | 5.05 | 0.01 | 1.28 | 2.03 | 0.04 | 5.67 | 0.01 |
| Enterobacteriaceae | 0.22 | 3.09 | 4.08 | 6.76 | 8.17 | 0.22 | 6.64 | 8.34 | 6.36 | 11.56 |
| Helicobacteraceae | 0.71 | 0.48 | 0.05 | 16.99*^,#^ | 4.62*^,#^ | 0.84 | 0.45 | 0.07 | 11.14 | 2.77 |
| AC160630_f | 0.01 | 0.03 | 1.91 | 5.80* | 0.58 | 0.02 | 0.03 | 4.23 | 4.33 | 0.57 |
| Rikenellaceae | 1.40 | 1.24 | 1.66 | 1.24 | 0.65 | 1.16 | 1.36 | 2.36 | 0.69 | 0.51 |
| Christensenellaceae | 0.37 | 0.38 | 0.35 | 1.70 | 0.01* | 0.31 | 0.13 | 0.39 | 1.34 | 0.01 |
| Desulfovibrionaceae | 0.00 | 0.01 | 0.07 | 0.41*^,#^ | 1.86*^,#^ | 0.01 | 0.01 | 0.15 | 0.18 | 0.95 |
| Erysipelotrichaceae | 0.26 | 0.90 | 0.33 | 0.39 | 0.20 | 0.20 | 1.44 | 0.38 | 0.36 | 0.16 |
| Porphyromonadaceae | 0.33 | 0.41 | 0.55 | 0.01* | 0.01* | 0.35 | 0.29 | 0.61 | 0.01 | 0.00 |
| Mycoplasmataceae | 0.68 | 0.54 | 0.00 | 0.00 | 0.76^#^ | 0.74 | 0.84 | 0.00 | 0.00 | 0.47 |
| Peptostreptococcaceae | 0.01 | 0.01 | 0.01 | 0.59 | 2.39*^,#^ | 0.01 | 0.02 | 0.02 | 0.78 | 0.96 |
| Peptococcaceae | 0.36 | 0.37 | 0.47 | 0.00*^,#^ | 0.00*^,#^ | 0.28 | 0.29 | 0.44 | 0.00 | 0.00 |
| Mogibacterium_f | 0.17 | 0.22 | 0.13 | 0.01* | 0.00* | 0.07 | 0.16 | 0.14 | 0.01 | 0.00 |

Con, Sha, Isc, IscA, and IscV in the x-axis of figures indicate groups treated with vehicle in normal control mice, vehicle in sham mice, vehicle in mice with Isc, ampicillin in mice with Isc, and vancomycin in mice with Isc, respectively. **p*<0.05 vs. Sha group, ^#^*p*<0.05 vs. Isc group.

**Supplement Table S2.** The gut microbiota composition ratio at the genus level in ischemia-induced mice treated with or without antibiotics

|  | **Average** | | | | | **Standard Deviation** | | | | |
| --- | --- | --- | --- | --- | --- | --- | --- | --- | --- | --- |
| **Taxon Name** | **Con** | **Sha** | **Isc** | **IscA** | **IscV** | **Con** | **Sha** | **Isc** | **IscA** | **IscV** |
| Bacteroides | 19.89 | 16.65 | 8.33 | 6.45 | 31.79^#^ | 13.50 | 9.46 | 11.59 | 5.29 | 17.45 |
| PAC000186_g | 15.16 | 12.40 | 7.27 | 6.01 | 12.68 | 7.50 | 8.00 | 5.48 | 4.46 | 4.10 |
| Helicobacter | 0.90 | 0.28 | 0.05 | 16.98*^,#^ | 4.62*^,#^ | 0.76 | 0.35 | 0.07 | 11.13 | 2.77 |
| PAC001068_g | 8.73 | 13.70 | 18.24 | 17.66 | 2.59*^,#^ | 2.75 | 7.87 | 13.94 | 3.61 | 0.87 |
| Blautia | 0.02 | 0.01 | 0.02 | 10.31 | 0.34*^,#^ | 0.02 | 0.01 | 0.03 | 13.76 | 0.18 |
| PAC001112_g | 5.29 | 2.98 | 8.66 | 5.31 | 4.17 | 2.14 | 1.82 | 5.89 | 1.73 | 0.55 |
| PAC001066_g | 0.47 | 1.18 | 1.45 | 0.95 | 5.72 | 0.34 | 0.88 | 1.04 | 0.17 | 6.63 |
| Paraprevotella | 4.01 | 0.90 | 0.02* | 0.01* | 0.02* | 3.83 | 0.66 | 0.03 | 0.01 | 0.02 |
| Parasutterella | 0.00 | 0.01 | 0.02 | 0.00 | 6.56*^,#^ | 0.00 | 0.01 | 0.04 | 0.00 | 1.75 |
| Escherichia | 0.20 | 3.10 | 0.32 | 5.34 | 3.90 | 0.23 | 6.63 | 0.31 | 6.01 | 4.67 |
| Muribaculum | 1.49 | 1.99 | 3.68 | 0.11*^,#^ | 5.25* | 1.21 | 0.95 | 2.95 | 0.10 | 0.94 |
| PAC002482_g | 0.02 | 0.02 | 1.91 | 5.79* | 0.58 | 0.03 | 0.03 | 4.22 | 4.33 | 0.57 |
| Clostridium_g24 | 0.26 | 0.27 | 0.14 | 0.25 | 3.46^#^ | 0.14 | 0.29 | 0.11 | 0.14 | 3.11 |
| Enterobacter | 0.00 | 0.00 | 3.72 | 0.01 | 4.06 | 0.01 | 0.00 | 8.31 | 0.01 | 7.90 |
| Enterobacteriaceae_g | 0.06 | 0.05 | 0.06 | 2.64*^,#^ | 3.74 | 0.08 | 0.07 | 0.09 | 1.58 | 7.97 |
| Prevotella | 3.99 | 2.63 | 3.38 | 0.01*^,#^ | 0.02*^,#^ | 1.80 | 2.47 | 2.80 | 0.01 | 0.02 |
| Pseudoflavonifractor | 2.43 | 2.40 | 1.31 | 0.42* | 0.01*^,#^ | 1.68 | 1.23 | 0.90 | 0.39 | 0.01 |
| Clostridium | 0.57 | 1.07 | 0.04 | 5.05 | 0.01 | 1.26 | 2.01 | 0.04 | 5.67 | 0.01 |
| Akkermansia | 2.94 | 4.86 | 3.23 | 0.01* | 2.04 | 3.25 | 4.37 | 4.45 | 0.01 | 1.20 |
| Lactobacillus | 0.50 | 3.17 | 0.43 | 0.14 | 0.35 | 0.43 | 5.02 | 0.42 | 0.06 | 0.11 |
| Alistipes | 1.44 | 1.19 | 1.66 | 1.24 | 0.65 | 1.14 | 1.37 | 2.36 | 0.69 | 0.51 |
| PAC000198_g | 2.34 | 3.11 | 5.05 | 2.76 | 0.01*^,#^ | 0.77 | 1.46 | 3.58 | 1.16 | 0.01 |
| HM123997_g | 1.16 | 3.02 | 2.31 | 0.02*^,#^ | 0.02*^,#^ | 2.55 | 2.41 | 1.34 | 0.02 | 0.02 |
| KE159538_g | 2.91 | 1.06 | 1.85 | 0.31 | 0.02* | 3.20 | 0.67 | 3.37 | 0.54 | 0.03 |
| Anaerotruncus | 1.27 | 0.28 | 0.17 | 0.36 | 0.04 | 1.34 | 0.28 | 0.20 | 0.28 | 0.04 |

Con, Sha, Isc, IscA, and IscV in the x-axis of figures indicate groups treated with vehicle in normal control mice, vehicle in sham mice, vehicle in mice with Isc, ampicillin in mice with Isc, and vancomycin in mice with Isc, respectively. **p*<0.05 vs. Sha group, ^#^*p*<0.05 vs. Isc group.

**Supplement Table S3.** The gut microbiota composition ratio at the family level in ischemia-induced mice treated with or without *Enterobacter xiangfangenesis*

|  | **Average** | | | | | **Standard Deviation** | | | | |
| --- | --- | --- | --- | --- | --- | --- | --- | --- | --- | --- |
| **Taxon Name** | **Con** | **Sha** | **Isc** | **Ex** | **IEx** | **Con** | **Sha** | **Isc** | **Ex** | **IEx** |
| Muribaculaceae | 44.71 | 46.96 | 47.77 | 17.21* | 23.56^#^ | 16.08 | 24.04 | 13.15 | 7.99 | 8.07 |
| Lachnospiraceae | 20.12 | 17.25 | 16.82 | 45.80* | 38.30^#^ | 16.16 | 15.80 | 8.51 | 5.42 | 15.37 |
| Ruminococcaceae | 8.63 | 7.24 | 10.28 | 10.63 | 8.19 | 5.02 | 4.36 | 4.71 | 3.16 | 3.58 |
| Bacteroidaceae | 11.03 | 9.11 | 7.66 | 3.07* | 7.32 | 4.64 | 5.34 | 3.27 | 1.57 | 7.28 |
| Rikenellaceae | 2.55 | 4.77 | 1.36 | 3.04 | 3.46 | 2.15 | 3.04 | 1.47 | 1.82 | 1.85 |
| Helicobacteraceae | 1.85 | 4.68 | 1.06 | 7.23* | 5.49^#^ | 1.80 | 7.41 | 0.86 | 2.61 | 2.73 |
| Deferribacteraceae | 1.57 | 0.92 | 1.21 | 0.64 | 0.29 | 2.49 | 1.88 | 2.19 | 0.35 | 0.15 |
| AC160630_f | 0.89 | 1.07 | 0.86 | 2.86* | 5.09*^,#^ | 1.05 | 0.73 | 0.55 | 0.61 | 3.79 |
| Lactobacillaceae | 3.85 | 1.60 | 5.85* | 0.08 | 0.30^#^ | 4.93 | 1.31 | 3.67 | 0.03 | 0.19 |
| Christensenellaceae | 0.43 | 0.72 | 1.29 | 0.69 | 1.22 | 0.32 | 0.52 | 0.95 | 0.37 | 1.05 |
| Odoribacteraceae | 0.38 | 0.56 | 0.02 | 0.70 | 0.60^#^ | 0.66 | 0.58 | 0.02 | 0.68 | 0.33 |
| FR888536_f | 0.41 | 0.76 | 0.27 | 0.15 | 0.17 | 0.37 | 0.68 | 0.24 | 0.12 | 0.11 |
| PAC000197_f | 0.15 | 0.23 | 0.32 | 0.08 | 0.07 | 0.20 | 0.20 | 0.39 | 0.05 | 0.04 |
| Prevotellaceae | 0.49 | 1.40 | 0.99 | 4.61 | 2.51 | 0.87 | 1.99 | 2.11 | 4.24 | 1.49 |
| Desulfovibrionaceae | 0.35 | 0.26 | 0.04* | 1.53* | 1.13*^,#^ | 0.22 | 0.16 | 0.04 | 0.77 | 0.76 |
| Porphyromonadaceae | 0.13 | 0.45 | 0.33 | 0.59* | 0.90 | 0.15 | 0.70 | 0.34 | 0.37 | 0.71 |
| Acholeplasmataceae | 0.05 | 0.00 | 0.01 | 0.08 | 0.52 | 0.11 | 0.00 | 0.01 | 0.07 | 0.67 |
| Clostridiaceae | 0.08 | 0.06 | 0.06 | 0.05 | 0.07 | 0.09 | 0.03 | 0.06 | 0.02 | 0.06 |
| Enterobacterales_f | 0.07 | 0.07 | 0.07 | 0.10 | 0.06 | 0.04 | 0.05 | 0.06 | 0.06 | 0.05 |
| Erysipelotrichaceae | 0.81 | 0.43 | 0.09 | 0.02 | 0.08 | 1.68 | 0.89 | 0.02 | 0.01 | 0.08 |

Con, Sha, Isc, Ex, and IEx in the x-axis of figures indicate groups treated with vehicle in normal control mice, vehicle in sham mice, vehicle in mice with Isc, *Enterobacter xiangfangenesis* in control mice, and *Enterobacter xiangfangenesis* in mice with Isc, respectively. **p*<0.05 vs. Con or Sha group, ^#^*p*<0.05 vs. Isc group.

**Supplement Table S4.** The gut microbiota composition ratio at the genus level in ischemia-induced mice treated with or without *Enterobacter xiangfangenesis*

|  | **Average** | | | | | **Standard Deviation** | | | | |
| --- | --- | --- | --- | --- | --- | --- | --- | --- | --- | --- |
| **Taxon Name** | **Con** | **Sha** | **Isc** | **Ex** | **IEx** | **Con** | **Sha** | **Isc** | **Ex** | **IEx** |
| Bacteroides | 11.02 | 9.11 | 7.66 | 3.07* | 7.32 | 4.63 | 5.34 | 3.27 | 1.57 | 7.28 |
| PAC001068_g | 8.98 | 11.30 | 8.15 | 5.39* | 5.18 | 2.08 | 7.66 | 4.08 | 2.77 | 1.65 |
| PAC000186_g | 5.42 | 6.18 | 7.45 | 1.52* | 1.84^#^ | 2.53 | 4.56 | 2.41 | 0.71 | 0.64 |
| PAC000664_g | 3.40 | 4.56 | 6.39 | 13.97* | 5.30 | 3.80 | 3.49 | 3.26 | 6.14 | 4.27 |
| Alistipes | 2.55 | 4.77 | 1.36 | 2.55 | 3.06 | 2.16 | 3.04 | 1.47 | 1.69 | 1.79 |
| HM123997_g | 0.34 | 0.46 | 0.02 | 0.72 | 1.39^#^ | 0.32 | 0.44 | 0.01 | 0.60 | 0.92 |
| Helicobacter | 1.85 | 4.68 | 1.06 | 7.23* | 5.49^#^ | 1.80 | 7.41 | 0.86 | 2.61 | 2.73 |
| KE159538_g | 2.56 | 3.08 | 1.61 | 7.16 | 7.57^#^ | 2.55 | 4.57 | 2.16 | 3.92 | 5.28 |
| Oscillibacter | 2.97 | 2.16 | 0.73 | 4.60 | 3.65^#^ | 3.43 | 2.24 | 0.72 | 2.65 | 1.94 |
| PAC001112_g | 7.69 | 7.07 | 8.16 | 1.26* | 1.45^#^ | 3.24 | 7.18 | 5.18 | 0.63 | 0.38 |
| LLKB_g | 2.34 | 1.57 | 0.18 | 3.76 | 3.58^#^ | 3.09 | 1.78 | 0.18 | 1.65 | 2.09 |
| Pseudoflavonifractor | 2.24 | 2.38 | 2.65 | 3.00 | 1.94 | 1.90 | 2.34 | 1.96 | 1.10 | 0.98 |
| KE159600_g | 2.12 | 0.71 | 0.46 | 1.69 | 2.51^#^ | 2.36 | 0.79 | 0.42 | 0.81 | 1.71 |
| Mucispirillum | 1.57 | 0.92 | 1.21 | 0.64 | 0.29 | 2.49 | 1.88 | 2.19 | 0.35 | 0.15 |
| PAC001074_g | 3.39 | 4.21 | 1.80 | 1.66 | 4.70 | 2.66 | 3.89 | 3.28 | 0.81 | 6.11 |
| PAC001485_g | 2.82 | 4.42 | 4.73 | 0.03* | 0.03*^,#^ | 1.06 | 3.35 | 3.02 | 0.01 | 0.02 |
| Muribaculum | 3.85 | 4.27 | 3.22 | 1.54 | 0.83^#^ | 4.07 | 5.03 | 1.33 | 0.89 | 0.54 |
| PAC001091_g | 0.61 | 0.49 | 0.95 | 1.79 | 1.78 | 1.16 | 0.48 | 0.85 | 1.36 | 1.37 |
| PAC002482_g | 0.89 | 1.07 | 0.86 | 2.86* | 5.07*^,#^ | 1.05 | 0.73 | 0.55 | 0.61 | 3.76 |
| Prevotella | 0.08 | 0.23 | 0.07 | 1.03 | 1.06*^,#^ | 0.05 | 0.15 | 0.11 | 1.03 | 0.52 |
| Alloprevotella | 0.00 | 0.00 | 0.00 | 0.38 | 0.26 | 0.00 | 0.00 | 0.00 | 0.48 | 0.48 |
| FR888536_g | 0.40 | 0.76 | 0.27 | 0.15 | 0.17 | 0.37 | 0.68 | 0.24 | 0.12 | 0.11 |
| HM630235_g | 0.00 | 0.00 | 0.00 | 0.29* | 0.23*^,#^ | 0.00 | 0.00 | 0.00 | 0.13 | 0.17 |
| Lactobacillus | 3.85 | 1.60 | 5.85* | 0.08 | 0.30^#^ | 4.93 | 1.31 | 3.66 | 0.03 | 0.19 |
| PAC002367_g | 0.04 | 0.42 | 1.45 | 2.24 | 1.64 | 0.03 | 0.66 | 3.03 | 2.35 | 2.42 |

Con, Sha, Isc, Ex, and IEx in the x-axis of figures indicate groups treated with vehicle in normal control mice, vehicle in sham mice, vehicle in mice with Isc, *Enterobacter xiangfangenesis* in control mice, and *Enterobacter xiangfangenesis* in mice with Isc, respectively. **p*<0.05 vs. Con or Sha group, ^#^*p*<0.05 vs. Isc group.

**Table S5.** Mean and SD of raw data in the experiments

| Fig 1. | | | | | | | |
| --- | --- | --- | --- | --- | --- | --- | --- |
|  | |  | Con | Sha | Isc | IscA | IscV |
| (B) (a) Spont. Alteration (%) | | Mean | 71 | 71 | 64 | 52 | 49 |
|  |  | SD | 1.69 | 2.54 | 6.96 | 5.47 | 6.48 |
| (b) Total distance moved (cm) | | Mean | 804 | 780 | 708 | 695 | 673 |
|  |  | SD | 106.24 | 94.87 | 126.68 | 99.79 | 113.68 |
| (C) % Time in novel | | Mean | 81 | 78 | 60 | 50 | 48 |
|  |  | SD | 1.60 | 3.86 | 3.11 | 4.57 | 5.79 |
| (D) Escape latency (s) | 1D | Mean | 43 | 43 | 81 | 149 | 128 |
|  |  | SD | 9.47 | 9.65 | 12.80 | 32.16 | 27.69 |
|  | 2D | Mean | 16 | 17 | 58 | 76 | 108 |
|  |  | SD | 9.37 | 12.05 | 11.40 | 24.56 | 31.09 |
|  | 3D | Mean | 13 | 15 | 41 | 68 | 97 |
|  |  | SD | 4.97 | 1.29 | 5.97 | 19.86 | 31.79 |
| (E) TNF-α (pg/mg) | | Mean | 5.8 | 6.2 | 6.8 | 13.7 | 15.0 |
|  |  | SD | 1.34 | 1.34 | 1.77 | 2.98 | 3.21 |
| (F) IL-1β (pg/mg) | | Mean | 31.0 | 51.5 | 119.3 | 291.2 | 303.7 |
|  |  | SD | 6.24 | 7.02 | 9.53 | 74.36 | 8.94 |
| (L) LPS (ng/mL) | | Mean | 4.8 | 5.7 | 19.0 | 41.7 | 46.3 |
|  |  | SD | 1.34 | 1.49 | 4.20 | 8.67 | 10.77 |
| (M) TNF-α (pg/mL) | | Mean | 5.8 | 6.2 | 6.8 | 17.2 | 16.0 |
|  |  | SD | 1.34 | 1.34 | 1.77 | 1.67 | 1.29 |

| Fig 2. | | | |
| --- | --- | --- | --- |
|  |  | Con | V |
| (A) Spont. Alteration (%) | Mean | 66.88 | 57.04 |
|  | SD | 5.20 | 9.88 |
| (D) IL-1β (pg/mg) | Mean | 3.67 | 8.00 |
|  | SD | 1.63 | 2.37 |

| Fig 3. | | | | | | |
| --- | --- | --- | --- | --- | --- | --- |
|  |  | Con | Sha | Isc | IscA | IscV |
| (A) Shannon | Mean | 3.85 | 3.91 | 4.17 | 3.17 | 3.09 |
|  | SD | 0.42 | 0.36 | 0.62 | 0.47 | 0.16 |
| (F) LPS (ng/mg) | Mean | 6 | 6 | 47 | 133 | 141 |
|  | SD | 1.70 | 1.91 | 5.20 | 30.28 | 36.99 |
| (G) Colon length (cm) | Mean | 5.8 | 5.8 | 5.4 | 4.9 | 5.0 |
|  | SD | 0.12 | 0.10 | 0.12 | 0.25 | 0.20 |
| (H) MPO activity (μUnit/mg) | Mean | 0.37 | 0.48 | 1.84 | 3.00 | 3.12 |
|  | SD | 0.10 | 0.17 | 0.31 | 0.52 | 0.52 |
| (I) IL-1β (pg/mg) | Mean | 33 | 34 | 68 | 216 | 214 |
|  | SD | 3.44 | 2.48 | 5.62 | 54.20 | 9.28 |
| (J) TNF-α (pg/mg) | Mean | 44 | 43 | 55 | 132 | 128 |
|  | SD | 2.34 | 8.50 | 3.42 | 4.11 | 6.72 |

| Fig 4. | | | | | | | |
| --- | --- | --- | --- | --- | --- | --- | --- |
|  |  | Con | Fcon | Isc | IFI | IFV | IFIV |
| (B) Spont. Alteration (%) | Mean | 72 | 70 | 56 | 54 | 47 | 47 |
|  | SD | 3.25 | 3.30 | 2.43 | 4.00 | 4.01 | 3.93 |
| (C) % Time in novel | Mean | 80 | 80 | 60 | 57 | 55 | 50 |
|  | SD | 1.49 | 1.34 | 3.09 | 5.25 | 3.20 | 3.62 |
| (H) Colon length (cm) | Mean | 5.8 | 5.8 | 5.4 | 5.3 | 5.2 | 5.2 |
|  | SD | 0.11 | 0.12 | 0.12 | 0.14 | 0.20 | 0.22 |
| (I) MPO activity (μUnit/mg) | Mean | 0.34 | 0.50 | 0.74 | 0.90 | 0.97 | 1.10 |
|  | SD | 0.06 | 0.12 | 0.09 | 0.11 | 0.11 | 0.16 |

| Fig 5 | | | | | | | |
| --- | --- | --- | --- | --- | --- | --- | --- |
|  | |  | Con | Sha | Isc | Ex | IEx |
| (B) Spont. Alteration (%) | | Mean | 71 | 70 | 55 | 51 | 47 |
|  |  | SD | 2.59 | 0.69 | 2.83 | 3.44 | 2.50 |
| (C) % Time in novel | | Mean | 81 | 79 | 60 | 55 | 39 |
|  |  | SD | 1.71 | 2.91 | 3.02 | 4.57 | 6.04 |
| (D) Escape latency (s) | 1D | Mean | 42 | 42 | 114 | 119 | 138 |
|  |  | SD | 6.29 | 9.12 | 21.83 | 29.58 | 40.43 |
|  | 2D | Mean | 22 | 20 | 52 | 49 | 105 |
|  |  | SD | 7.55 | 8.72 | 6.62 | 7.87 | 11.12 |
|  | 3D | Mean | 13 | 10 | 25 | 25 | 102 |
|  |  | SD | 4.27 | 4.92 | 6.53 | 6.40 | 20.66 |
| (E) TNF-α (pg/mg) | | Mean | 6 | 6 | 16 | 17 | 20 |
|  |  | SD | 1.63 | 1.91 | 0.82 | 2.89 | 1.97 |
| (F) IL-1β (pg/mg) | | Mean | 25 | 23 | 56 | 55 | 67 |
|  |  | SD | 2.21 | 4.10 | 3.24 | 4.53 | 9.81 |
| (L) LPS (ng/mL) | | Mean | 13 | 17 | 28 | 70 | 72 |
|  |  | SD | 3.24 | 2.99 | 5.91 | 6.41 | 5.15 |
| (M) TNF-α (pg/mL) | | Mean | 4.2 | 4.0 | 4.5 | 5.2 | 7.2 |
|  |  | SD | 1.07 | 1.41 | 0.76 | 1.07 | 1.34 |

| Fig 6. | | | | | | |
| --- | --- | --- | --- | --- | --- | --- |
|  |  | Con | Sha | Isc | Ex | IEx |
| (A) Shannon | Mean | 4.28 | 4.13 | 4.26 | 4.75 | 4.81 |
|  | SD | 0.16 | 0.26 | 0.24 | 0.11 | 0.19 |
| (E) LPS (ng/mg) | Mean | 6 | 7 | 23 | 194 | 201 |
|  | SD | 1.80 | 2.49 | 5.85 | 28.69 | 39.20 |
| (F) Colon length (cm) | Mean | 5.8 | 5.7 | 5.4 | 5.2 | 5.0 |
|  | SD | 0.13 | 0.11 | 0.15 | 0.21 | 0.15 |
| (G) MPO activity (μUnit/mg) | Mean | 0.34 | 0.49 | 1.13 | 7.10 | 7.43 |
|  | SD | 0.08 | 0.11 | 0.41 | 0.54 | 0.83 |
| (H) TNF-α (pg/mg) | Mean | 0.6 | 1.1 | 1.8 | 5.1 | 5.2 |
|  | SD | 0.24 | 0.35 | 0.66 | 0.59 | 0.34 |
| (I) IL-1β (pg/mg) | Mean | 48 | 50 | 53 | 52 | 54 |
|  | SD | 6.77 | 1.86 | 3.06 | 5.60 | 2.50 |

**Supplement Table S6**. P-values in the experiments

| Figure 1.  Bonferroni's multiple comparisons one-way ANOVA test (B, C, D, E, F, L, M) | | | | | | | | | | | | | |
| --- | --- | --- | --- | --- | --- | --- | --- | --- | --- | --- | --- | --- | --- |
| (B)  (a) Spont. Alteration (%)  F (DFn, DFd)  F (4, 25) = 21.10 | | Con vs. Sha | | | p>.9999 | | | | Sha vs. IscA | | | | p<0.0001 |
|  |  | Con vs. Isc | | | P=0.2421 | | | | Sha vs. IscV | | | | p<0.0001 |
|  |  | Con vs. IscA | | | p<0.0001 | | | | Isc vs. IscA | | | | P=0.0093 |
|  |  | Con vs. IscV | | | p<0.0001 | | | | Isc vs. IscV | | | | P=0.0012 |
|  |  | Sha vs. Isc | | | P=0.4244 | | | | IscA vs. IscV | | | | p>0.9999 |
| (b) Total distance moved (cm)  F (DFn, DFd)  F (4, 25) = 1.365 | | Con vs. Sha | | | p>0.9999 | | | | Sha vs. IscA | | | | p>0.9999 |
|  |  | Con vs. Isc | | | p>0.9999 | | | | Sha vs. IscV | | | | p>0.9999 |
|  |  | Con vs. IscA | | | p>0.9999 | | | | Isc vs. IscA | | | | p>0.9999 |
|  |  | Con vs. IscV | | | P=0.6823 | | | | Isc vs. IscV | | | | p>0.9999 |
|  |  | Sha vs. Isc | | | p>0.9999 | | | | IscA vs. IscV | | | | p>0.9999 |
| (C) % Time in novel  F (DFn, DFd)  F (4, 25) = 71.11 | | Con vs. Sha | | | p>0.9999 | | | | Sha vs. IscA | | | | p<0.0001 |
|  |  | Con vs. Isc | | | p<0.0001 | | | | Sha vs. IscV | | | | p<0.0001 |
|  |  | Con vs. IscA | | | p<0.0001 | | | | Isc vs. IscA | | | | P=0.0037 |
|  |  | Con vs. IscV | | | p<0.0001 | | | | Isc vs. IscV | | | | P=0.0011 |
|  |  | Sha vs. Isc | | | p<0.0001 | | | | IscA vs. IscV | | | | p>0.9999 |
| (D) Escape latency (s) | |  | | |  | | | |  | | | |  |
| 1D  F (DFn, DFd)  F (4, 25) = 27.43 | | Con vs. Sha | | | p>0.9999 | | | | Sha vs. IscA | | | | p<0.0001 |
|  |  | Con vs. Isc | | | P=0.0724 | | | | Sha vs. IscV | | | | p<0.0001 |
|  |  | Con vs. IscA | | | p<0.0001 | | | | Isc vs. IscA | | | | P=0.0002 |
|  |  | Con vs. IscV | | | p<0.0001 | | | | Isc vs. IscV | | | | P=0.0167 |
|  |  | Sha vs. Isc | | | P=0.0702 | | | | IscA vs. IscV | | | | p>0.9999 |
| 2D  F (DFn, DFd)  F (4, 25) = 20.03 | | Con vs. Sha | | | p>0.9999 | | | | Sha vs. IscA | | | | P=0.0007 |
|  |  | Con vs. Isc | | | P=0.0232 | | | | Sha vs. IscV | | | | p<0.0001 |
|  |  | Con vs. IscA | | | P=0.0005 | | | | Isc vs. IscA | | | | p>0.9999 |
|  |  | Con vs. IscV | | | p<0.0001 | | | | Isc vs. IscV | | | | P=0.0050 |
|  |  | Sha vs. Isc | | | P=0.0302 | | | | IscA vs. IscV | | | | P=0.1795 |
| 3D  F (DFn, DFd)  F (4, 25) = 22.13 | | Con vs. Sha | | | p>0.9999 | | | | Sha vs. IscA | | | | P=0.0005 |
|  |  | Con vs. Isc | | | P=0.1774 | | | | Sha vs. IscV | | | | p<0.0001 |
|  |  | Con vs. IscA | | | P=0.0003 | | | | Isc vs. IscA | | | | P=0.1654 |
|  |  | Con vs. IscV | | | p<0.0001 | | | | Isc vs. IscV | | | | P=0.0002 |
|  |  | Sha vs. Isc | | | P=0.2674 | | | | IscA vs. IscV | | | | P=0.1339 |
| (E) TNF-α (pg/mg)  F (DFn, DFd)  F (4, 25) = 19.08 | | Con vs. Sha | | | p>0.9999 | | | | Sha vs. IscA | | | | P=0.0002 |
|  |  | Con vs. Isc | | | p>0.9999 | | | | Sha vs. IscV | | | | p<0.0001 |
|  |  | Con vs. IscA | | | P=0.0001 | | | | Isc vs. IscA | | | | P=0.0007 |
|  |  | Con vs. IscV | | | p<0.0001 | | | | Isc vs. IscV | | | | p<0.0001 |
|  |  | Sha vs. Isc | | | p>0.9999 | | | | IscA vs. IscV | | | | p>0.9999 |
| (F) IL-1β (pg/mg)  F (DFn, DFd)  F (4, 25) = 73.33 | | Con vs. Sha | | | p>0.9999 | | | | Sha vs. IscA | | | | p<0.0001 |
|  |  | Con vs. Isc | | | P=0.0038 | | | | Sha vs. IscV | | | | p<0.0001 |
|  |  | Con vs. IscA | | | p<0.0001 | | | | Isc vs. IscA | | | | p<0.0001 |
|  |  | Con vs. IscV | | | p<0.0001 | | | | Isc vs. IscV | | | | p<0.0001 |
|  |  | Sha vs. Isc | | | P=0.0418 | | | | IscA vs. IscV | | | | p>0.9999 |
| (L) LPS (ng/mL)  F (DFn, DFd)  F (4, 25) = 45.17 | | Con vs. Sha | | | p>0.9999 | | | | Sha vs. IscA | | | | p<0.0001 |
|  |  | Con vs. Isc | | | P=0.0209 | | | | Sha vs. IscV | | | | p<0.0001 |
|  |  | Con vs. IscA | | | p<0.0001 | | | | Isc vs. IscA | | | | P=0.0001 |
|  |  | Con vs. IscV | | | p<0.0001 | | | | Isc vs. IscV | | | | p<0.0001 |
|  |  | Sha vs. Isc | | | P=0.0344 | | | | IscA vs. IscV | | | | p>0.9999 |
| (M) TNF-α (pg/mL)  F (DFn, DFd)  F (4, 25) = 71.65 | | Con vs. Sha | | | p>0.9999 | | | | Sha vs. IscA | | | | p<0.0001 |
|  |  | Con vs. Isc | | | p>0.9999 | | | | Sha vs. IscV | | | | p<0.0001 |
|  |  | Con vs. IscA | | | p<0.0001 | | | | Isc vs. IscA | | | | p<0.0001 |
|  |  | Con vs. IscV | | | p<0.0001 | | | | Isc vs. IscV | | | | p<0.0001 |
|  |  | Sha vs. Isc | | | p>0.9999 | | | | IscA vs. IscV | | | | p>0.9999 |
| Figure 2.  Two tailed Mann-Whitney U test | | | | | | | | | | | | | |
|  | | U statistic | | | Sum of ranks | | | | P value | | | |  |
|  | |  | | | Con | | | | V | | | |  |
| (A) Spont. Alteration (%) | | 5 | | | 52 | | | | 26 | | | | P=0.0346 |
| (D) IL-1β (pg/mg) | | 2 | | | 23 | | | | 55 | | | | P=0.0108 |
| Figure 3.  Bonferroni's multiple comparisons one-way ANOVA test (A, G, H, J)  Holm-Sidak's multiple comparisons one-way ANOVA test (F, I) | | | | | | | | | | | | | |
| (A) Shannon  F (DFn, DFd)  F (4, 20) = 4.893 | Con vs. Sha | | | p>0.9999 | | | Sha vs. IscA | | | | P=0.0487 | | |
|  | Con vs. Isc | | | p>0.9999 | | | Sha vs. IscV | | | | P=0.0478 | | |
|  | Con vs. IscA | | | P=0.0779 | | | Isc vs. IscA | | | | P=0.0394 | | |
|  | Con vs. IscV | | | P=0.0890 | | | Isc vs. IscV | | | | P=0.0216 | | |
|  | Sha vs. Isc | | | p>0.9999 | | | IscA vs. IscV | | | | p>0.9999 | | |
| (F) LPS (ng/mg)  F (DFn, DFd)  F (4, 25) = 47.50 | Con vs. Sha | | | P=0.9807 | | | Sha vs. IscA | | | | p<0.0001 | | |
|  | Con vs. Isc | | | P=0.0220 | | | Sha vs. IscV | | | | p<0.0001 | | |
|  | Con vs. IscA | | | p<0.0001 | | | Isc vs. IscA | | | | p<0.0001 | | |
|  | Con vs. IscV | | | p<0.0001 | | | Isc vs. IscV | | | | p<0.0001 | | |
|  | Sha vs. Isc | | | P=0.0220 | | | IscA vs. IscV | | | | P=0.7940 | | |
| (G) Colon length (cm)  F (DFn, DFd)  F (4, 25) = 29.88 | Con vs. Sha | | | p>0.9999 | | | Sha vs. IscA | | | | p<0.0001 | | |
|  | Con vs. Isc | | | P=0.0178 | | | Sha vs. IscV | | | | p<0.0001 | | |
|  | Con vs. IscA | | | p<0.0001 | | | Isc vs. IscA | | | | P=0.0010 | | |
|  | Con vs. IscV | | | p<0.0001 | | | Isc vs. IscV | | | | P=0.0053 | | |
|  | Sha vs. Isc | | | P=0.0265 | | | IscA vs. IscV | | | | p>0.9999 | | |
| (H) MPO activity (μUnit/mg)  F (DFn, DFd)  F (4, 25) = 64.73 | Con vs. Sha | | | p>0.9999 | | | Sha vs. IscA | | | | p<0.0001 | | |
|  | Con vs. Isc | | | p<0.0001 | | | Sha vs. IscV | | | | p<0.0001 | | |
|  | Con vs. IscA | | | p<0.0001 | | | Isc vs. IscA | | | | P=0.0004 | | |
|  | Con vs. IscV | | | p<0.0001 | | | Isc vs. IscV | | | | p<0.0001 | | |
|  | Sha vs. Isc | | | p<0.0001 | | | IscA vs. IscV | | | | p>0.9999 | | |
| (I) IL-1β (pg/mg)  F (DFn, DFd)  F (4, 25) = 72.12 | Con vs. Sha | | | P=0.9930 | | | Sha vs. IscA | | | | p<0.0001 | | |
|  | Con vs. Isc | | | P=0.0440 | | | Sha vs. IscV | | | | p<0.0001 | | |
|  | Con vs. IscA | | | p<0.0001 | | | Isc vs. IscA | | | | p<0.0001 | | |
|  | Con vs. IscV | | | p<0.0001 | | | Isc vs. IscV | | | | p<0.0001 | | |
|  | Sha vs. Isc | | | P=0.0440 | | | IscA vs. IscV | | | | P=0.9930 | | |
| (J) TNF-α (pg/mg)  F (DFn, DFd)  F (4, 25) = 342.8 | Con vs. Sha | | | p>0.9999 | | | Sha vs. IscA | | | | p<0.0001 | | |
|  | Con vs. Isc | | | P=0.0560 | | | Sha vs. IscV | | | | p<0.0001 | | |
|  | Con vs. IscA | | | p<0.0001 | | | Isc vs. IscA | | | | p<0.0001 | | |
|  | Con vs. IscV | | | p<0.0001 | | | Isc vs. IscV | | | | p<0.0001 | | |
|  | Sha vs. Isc | | | P=0.0540 | | | IscA vs. IscV | | | | p>0.9999 | | |
| Figure 4.  Bonferroni's multiple comparisons one-way ANOVA test (B, C, H, I) | | | | | | | | | | | | | |
| (B) Spont. Alteration (%)  F (DFn, DFd)  F (5, 30) = 47.54 | | | Con vs. FCon | | | p>0.9999 | | FCon vs. IFIV | | | | p<0.0001 | |
|  |  |  | Con vs. Isc | | | p<0.0001 | | Isc vs. IFI | | | | p>0.9999 | |
|  |  |  | Con vs. IFI | | | p<0.0001 | | Isc vs. IFV | | | | P=0.0041 | |
|  |  |  | Con vs. IFV | | | p<0.0001 | | Isc vs. IFIV | | | | P=0.0043 | |
|  |  |  | Con vs. IFIV | | | p<0.0001 | | IFI vs. IFV | | | | P=0.0886 | |
|  |  |  | FCon vs. Isc | | | p<0.0001 | | IFI vs. IFIV | | | | P=0.0922 | |
|  |  |  | FCon vs. IFI | | | p<0.0001 | | IFV vs. IFIV | | | | p>0.9999 | |
|  |  |  | FCon vs. IFV | | | p<0.0001 | |  | | | |  | |
| (C) % Time in novel  F (DFn, DFd)  F (5, 30) = 80.16 | | | Con vs. FCon | | | p>0.9999 | | FCon vs. IFIV | | | | p<0.0001 | |
|  |  |  | Con vs. Isc | | | p<0.0001 | | Isc vs. IFI | | | | p>0.9999 | |
|  |  |  | Con vs. IFI | | | p<0.0001 | | Isc vs. IFV | | | | P=0.3335 | |
|  |  |  | Con vs. IFV | | | p<0.0001 | | Isc vs. IFIV | | | | P=0.0003 | |
|  |  |  | Con vs. IFIV | | | p<0.0001 | | IFI vs. IFV | | | | p>0.9999 | |
|  |  |  | FCon vs. Isc | | | p<0.0001 | | IFI vs. IFIV | | | | P=0.0162 | |
|  |  |  | FCon vs. IFI | | | p<0.0001 | | IFV vs. IFIV | | | | P=0.1898 | |
|  |  |  | FCon vs. IFV | | | p<0.0001 | |  | | | |  | |
| (H) Colon length (cm)  F (DFn, DFd)  F (5, 30) = 16.75 | | | Con vs. FCon | | | p>0.9999 | | FCon vs. IFIV | | | | p<0.0001 | |
|  |  |  | Con vs. Isc | | | P=0.0027 | | Isc vs. IFI | | | | p>0.9999 | |
|  |  |  | Con vs. IFI | | | P=0.0002 | | Isc vs. IFV | | | | p>0.9999 | |
|  |  |  | Con vs. IFV | | | p<0.0001 | | Isc vs. IFIV | | | | P=0.7380 | |
|  |  |  | Con vs. IFIV | | | p<0.0001 | | IFI vs. IFV | | | | p>0.9999 | |
|  |  |  | FCon vs. Isc | | | P=0.0043 | | IFI vs. IFIV | | | | p>0.9999 | |
|  |  |  | FCon vs. IFI | | | P=0.0002 | | IFV vs. IFIV | | | | p>0.9999 | |
|  |  |  | FCon vs. IFV | | | p<0.0001 | |  | | | |  | |
| (I) MPO activity (μUnit/mg)  F (DFn, DFd)  F (5, 30) = 32.71 | | | Con vs. FCon | | | P=0.4606 | | FCon vs. IFIV | | | | P<0.0001 | |
|  |  |  | Con vs. Isc | | | p<0.0001 | | Isc vs. IFI | | | | P=0.5346 | |
|  |  |  | Con vs. IFI | | | p<0.0001 | | Isc vs. IFV | | | | P=0.0653 | |
|  |  |  | Con vs. IFV | | | p<0.0001 | | Isc vs. IFIV | | | | P=0.0003 | |
|  |  |  | Con vs. IFIV | | | p<0.0001 | | IFI vs. IFV | | | | p>0.9999 | |
|  |  |  | FCon vs. Isc | | | P=0.0507 | | IFI vs. IFIV | | | | P=0.1132 | |
|  |  |  | FCon vs. IFI | | | p<0.0001 | | IFV vs. IFIV | | | | p>0.9999 | |
|  |  |  | FCon vs. IFV | | | p<0.0001 | |  | | | |  | |
| Figure 5.  Bonferroni's multiple comparisons one-way ANOVA test (B, C, D, F, L, M) | | | | | | | | | | | | | |
| (B) Spont. Alteration (%)  F (DFn, DFd)  F (4, 25) = 95.86 | | | Con vs. Sha | | | p>0.9999 | | | | Sha vs. Ex | | p<0.0001 | |
|  |  |  | Con vs. Isc | | | p<0.0001 | | | | Sha vs. IEx | | p<0.0001 | |
|  |  |  | Con vs. Ex | | | p<0.0001 | | | | Isc vs. Ex | | P=0.1926 | |
|  |  |  | Con vs. IEx | | | p<0.0001 | | | | Isc vs. IEx | | P=0.0002 | |
|  |  |  | Sha vs. Isc | | | p<0.0001 | | | | Ex vs. IEx | | P=0.1360 | |
| (C) % Time in novel  F (DFn, DFd)  F (4, 25) = 96.76 | | | Con vs. Sha | | | p>0.9999 | | | | Sha vs. Ex | | p<0.0001 | |
|  |  |  | Con vs. Isc | | | p<0.0001 | | | | Sha vs. IEx | | p<0.0001 | |
|  |  |  | Con vs. Ex | | | p<0.0001 | | | | Isc vs. Ex | | P=0.4264 | |
|  |  |  | Con vs. IEx | | | p<0.0001 | | | | Isc vs. IEx | | p<0.0001 | |
|  |  |  | Sha vs. Isc | | | p<0.0001 | | | | Ex vs. IEx | | p<0.0001 | |
| (D) Escape latency (s) | | |  | | |  | | | |  | |  | |
| 1D  F (DFn, DFd)  F (4, 25) = 16.82 | | | Con vs. Sha | | | p>0.9999 | | | | Sha vs. Ex | | P=0.0005 | |
|  |  |  | Con vs. Isc | | | P=0.0011 | | | | Sha vs. IEx | | p<0.0001 | |
|  |  |  | Con vs. Ex | | | P=0.0005 | | | | Isc vs. Ex | | p>0.9999 | |
|  |  |  | Con vs. IEx | | | p<0.0001 | | | | Isc vs. IEx | | p>0.9999 | |
|  |  |  | Sha vs. Isc | | | P=0.0012 | | | | Ex vs. IEx | | p>0.9999 | |
| 2D  F (DFn, DFd)  F (4, 25) = 81.92 | | | Con vs. Sha | | | p>0.9999 | | | | Sha vs. Ex | | P=0.0002 | |
|  |  |  | Con vs. Isc | | | P=0.0001 | | | | Sha vs. IEx | | p<0.0001 | |
|  |  |  | Con vs. Ex | | | P=0.0005 | | | | Isc vs. Ex | | p>0.9999 | |
|  |  |  | Con vs. IEx | | | p<0.0001 | | | | Isc vs. IEx | | p<0.0001 | |
|  |  |  | Sha vs. Isc | | | p<0.0001 | | | | Ex vs. IEx | | p<0.0001 | |
| 3D  F (DFn, DFd)  F (4, 25) = 64.80 | | | Con vs. Sha | | | p>0.9999 | | | | Sha vs. Ex | | p>0.9999 | |
|  |  |  | Con vs. Isc | | | P=0.3686 | | | | Sha vs. IEx | | p<0.0001 | |
|  |  |  | Con vs. Ex | | | P=0.4319 | | | | Isc vs. Ex | | p>0.9999 | |
|  |  |  | Con vs. IEx | | | p<0.0001 | | | | Isc vs. IEx | | p<0.0001 | |
|  |  |  | Sha vs. Isc | | | P=0.9165 | | | | Ex vs. IEx | | p<0.0001 | |
| (E) TNF-α (pg/mg)  F (DFn, DFd)  F (4, 25) = 54.44 | | | Con vs. Sha | | | p>0.9999 | | | | Sha vs. Ex | | p<0.0001 | |
|  |  |  | Con vs. Isc | | | p<0.0001 | | | | Sha vs. IEx | | p<0.0001 | |
|  |  |  | Con vs. Ex | | | p<0.0001 | | | | Isc vs. Ex | | p>0.9999 | |
|  |  |  | Con vs. IEx | | | p<0.0001 | | | | Isc vs. IEx | | P=0.0470 | |
|  |  |  | Sha vs. Isc | | | p<0.0001 | | | | Ex vs. IEx | | P=0.4139 | |
| (F) IL-1β (pg/mg)  F (DFn, DFd)  F (4, 25) = 65.62 | | | Con vs. Sha | | | p>0.9999 | | | | Sha vs. Ex | | p<0.0001 | |
|  |  |  | Con vs. Isc | | | p<0.0001 | | | | Sha vs. IEx | | p<0.0001 | |
|  |  |  | Con vs. Ex | | | p<0.0001 | | | | Isc vs. Ex | | p>0.9999 | |
|  |  |  | Con vs. IEx | | | p<0.0001 | | | | Isc vs. IEx | | P=0.0646 | |
|  |  |  | Sha vs. Isc | | | p<0.0001 | | | | Ex vs. IEx | | P=0.0503 | |
| (L) LPS (ng/mL)  F (DFn, DFd)  F (4, 25) = 173.8 | | | Con vs. Sha | | | p>0.9999 | | | | Sha vs. Ex | | p<0.0001 | |
|  |  |  | Con vs. Isc | | | P=0.0008 | | | | Sha vs. IEx | | p<0.0001 | |
|  |  |  | Con vs. Ex | | | p<0.0001 | | | | Isc vs. Ex | | p<0.0001 | |
|  |  |  | Con vs. IEx | | | p<0.0001 | | | | Isc vs. IEx | | p<0.0001 | |
|  |  |  | Sha vs. Isc | | | P=0.0167 | | | | Ex vs. IEx | | p>0.9999 | |
| (M) TNF-α (pg/mL)  F (DFn, DFd)  F (4, 25) = 6.250 | | | Con vs. Sha | | | p>0.9999 | | | | Sha vs. Ex | | p>0.9999 | |
|  |  |  | Con vs. Isc | | | p>0.9999 | | | | Sha vs. IEx | | P=0.0021 | |
|  |  |  | Con vs. Ex | | | p>0.9999 | | | | Isc vs. Ex | | p>0.9999 | |
|  |  |  | Con vs. IEx | | | P=0.0038 | | | | Isc vs. IEx | | P=0.0121 | |
|  |  |  | Sha vs. Isc | | | p>0.9999 | | | | Ex vs. IEx | | P=0.1121 | |
| Figure 6.  Bonferroni's multiple comparisons one-way ANOVA test (A, E, F, G, H, I) | | | | | | | | | | | | | |
| (A) Shannon  F (DFn, DFd)  F (4, 20) = 9.746 | | | Con vs. Sha | | | p>0.9999 | | | | Sha vs. Ex | | P=0.0028 | |
|  |  |  | Con vs. Isc | | | p>0.9999 | | | | Sha vs. IEx | | P=0.0011 | |
|  |  |  | Con vs. Ex | | | P=0.0324 | | | | Isc vs. Ex | | P=0.0226 | |
|  |  |  | Con vs. IEx | | | P=0.0121 | | | | Isc vs. IEx | | P=0.0084 | |
|  |  |  | Sha vs. Isc | | | p>0.9999 | | | | Ex vs. IEx | | p>0.9999 | |
| (E) LPS (ng/mg)  F (DFn, DFd)  F (4, 25) = 107.8 | | | Con vs. Sha | | | p>0.9999 | | | | Sha vs. Ex | | p<0.0001 | |
|  |  |  | Con vs. Isc | | | p>0.9999 | | | | Sha vs. IEx | | p<0.0001 | |
|  |  |  | Con vs. Ex | | | p<0.0001 | | | | Isc vs. Ex | | p<0.0001 | |
|  |  |  | Con vs. IEx | | | p<0.0001 | | | | Isc vs. IEx | | p<0.0001 | |
|  |  |  | Sha vs. Isc | | | p>0.9999 | | | | Ex vs. IEx | | p>0.9999 | |
| (F) Colon length (cm)  F (DFn, DFd)  F (4, 25) = 20.97 | | | Con vs. Sha | | | p>0.9999 | | | | Sha vs. Ex | | P=0.0002 | |
|  |  |  | Con vs. Isc | | | P=0.0053 | | | | Sha vs. IEx | | p<0.0001 | |
|  |  |  | Con vs. Ex | | | p<0.0001 | | | | Isc vs. Ex | | p>0.9999 | |
|  |  |  | Con vs. IEx | | | p<0.0001 | | | | Isc vs. IEx | | P=0.0198 | |
|  |  |  | Sha vs. Isc | | | P=0.0128 | | | | Ex vs. IEx | | P=0.6903 | |
| (G) MPO activity (μUnit/mg)  F (DFn, DFd)  F (4, 25) = 284.8 | | | Con vs. Sha | | | p>0.9999 | | | | Sha vs. Ex | | p<0.0001 | |
|  |  |  | Con vs. Isc | | | P=0.1669 | | | | Sha vs. IEx | | p<0.0001 | |
|  |  |  | Con vs. Ex | | | p<0.0001 | | | | Isc vs. Ex | | p<0.0001 | |
|  |  |  | Con vs. IEx | | | p<0.0001 | | | | Isc vs. IEx | | p<0.0001 | |
|  |  |  | Sha vs. Isc | | | P=0.4659 | | | | Ex vs. IEx | | p>0.9999 | |
| (H) TNF-α (pg/mg)  F (DFn, DFd)  F (4, 25) = 116.9 | | | Con vs. Sha | | | p>0.9999 | | | | Sha vs. Ex | | p<0.0001 | |
|  |  |  | Con vs. Isc | | | P=0.0034 | | | | Sha vs. IEx | | p<0.0001 | |
|  |  |  | Con vs. Ex | | | p<0.0001 | | | | Isc vs. Ex | | p<0.0001 | |
|  |  |  | Con vs. IEx | | | p<0.0001 | | | | Isc vs. IEx | | p<0.0001 | |
|  |  |  | Sha vs. Isc | | | P=0.1490 | | | | Ex vs. IEx | | p>0.9999 | |
| (I) IL-1β (pg/mg)  F (DFn, DFd)  F (4, 25) = 1.750 | | | Con vs. Sha | | | p>0.9999 | | | | Sha vs. Ex | | p>0.9999 | |
|  |  |  | Con vs. Isc | | | P=0.5650 | | | | Sha vs. IEx | | p>0.9999 | |
|  |  |  | Con vs. Ex | | | p>0.9999 | | | | Isc vs. Ex | | p>0.9999 | |
|  |  |  | Con vs. IEx | | | P=0.2744 | | | | Isc vs. IEx | | p>0.9999 | |
|  |  |  | Sha vs. Isc | | | p>0.9999 | | | | Ex vs. IEx | | p>0.9999 | |
